# Identified in blood diet-related methylation changes stratify liver biopsies of NAFLD patients according to fibrosis grade

**DOI:** 10.1186/s13148-022-01377-6

**Published:** 2022-11-30

**Authors:** Katarzyna Ewa Sokolowska, Dominika Maciejewska-Markiewicz, Jan Bińkowski, Joanna Palma, Olga Taryma-Leśniak, Katarzyna Kozlowska-Petriczko, Konrad Borowski, Magdalena Baśkiewicz-Hałasa, Viktoria Hawryłkowicz, Patrycja Załęcka, Marcin Ufnal, Dominik Strapagiel, Justyna Jarczak, Karolina Skonieczna-Żydecka, Karina Ryterska, Bogusław Machaliński, Tomasz Kazimierz Wojdacz, Ewa Stachowska

**Affiliations:** 1 Independent Clinical Epigenetics Laboratory, Pomeranian Medical University, Unii Lubelskiej 1, 71-252 Szczecin, Poland; 2 Department of Human Nutrition and Metabolomics, Pomeranian Medical University, 71-460 Szczecin, Poland; 3 Department of Biochemical Sciences, Pomeranian Medical University, 71-460 Szczecin, Poland; 4 Translational Medicine Group, Pomeranian Medical University, 70-204 Szczecin, Poland; 5 Department of Gastroenterology and Internal Medicine, SPWSZ Hospital, 71-455 Szczecin, Poland; 6 Department of General Pathology, Pomeranian Medical University, 70-111 Szczecin, Poland; 7 Department of Experimental Physiology and Pathophysiology, Laboratory of Centre for Preclinical Research, Medical University of Warsaw, 02-097 Warsaw, Poland; 8 Biobank Laboratory, Department of Molecular Biophysics, Faculty of Biology and Environmental Protection, University of Lodz, 90-237 Lodz, Poland; 9 Laboratory of Molecular Basis of Behavior, Nencki Institute of Experimental Biology, Polish Academy of Science, Warsaw, Poland

**Keywords:** Nutrition, Non-alcoholic fatty liver disease, Liver, Fibre, DNA methylation, Epigenetics, Acetylation, Mediterranean diet

## Abstract

**Background:**

High caloric diet and lack of physical activity are considered main causes of NAFLD, and a change in the diet is still the only effective treatment of this disease. However, molecular mechanism of the effectiveness of diet change in treatment of NAFLD is poorly understood. We aimed to assess the involvement of epigenetic mechanisms of gene expression regulation in treatment of NAFLD. Eighteen participants with medium- to high-grade steatosis were recruited and trained to follow the Mediterranean diet modified to include fibre supplements. At three timepoints (baseline, after 30 and 60 days), we evaluated adherence to the diet and measured a number of physiological parameters such as anthropometry, blood and stool biochemistry, liver steatosis and stiffness. We also collected whole blood samples for genome-wide methylation profiling and histone acetylation assessment.

**Results:**

The diet change resulted in a decrease in liver steatosis along with statistically significant, but a minor change in BMI and weight of our study participants. The epigenetic profiling of blood cells identified significant genome-wide changes of methylation and acetylation with the former not involving regions directly regulating gene expression. Most importantly, we were able to show that identified blood methylation changes occur also in liver cells of NAFLD patients and the machine learning-based classifier that we build on those methylation changes was able to predict the stage of liver fibrosis with ROC AUC = 0.9834.

**Conclusion:**

Methylomes of blood cells from NAFLD patients display a number of changes that are most likely a consequence of unhealthy diet, and the diet change appears to reverse those epigenetic changes. Moreover, the methylation status at CpG sites undergoing diet-related methylation change in blood cells stratifies liver biopsies from NAFLD patients according to fibrosis grade.

**Supplementary Information:**

The online version contains supplementary material available at 10.1186/s13148-022-01377-6.

## Background

Caloric access and decreasing physical activity is a leading cause of epidemic of obesity and metabolic syndrome. The comorbidity associated with this lifestyle is non-alcoholic fatty liver disease (NAFLD). The prevalence of NAFLD in general population has been shown to range from 11.2 to 37.2% and increase [1–3]. NAFLD is characterized by the presence of hepatic steatosis (diagnosed either by imagining or by histology) that cannot be attributed to excessive alcohol consumption. This condition leads to lipotoxicity and liver damage and, in the long term, significantly increases the risk of progression to steatohepatitis, cirrhosis and even to hepatocellular carcinoma (HCC) [4–6].

On cellular level, NAFLD is a consequence of the accumulation of the lipid droplets (LD) in hepatocytes, of which catabolism initiated normally by nutrient deprivation or acute LD overload is impaired. Quite a large number of medications and new compounds are in the I–III phase of clinical trials for NAFLD and NASH [7]. However, apart from the use of pioglitazone and vitamin E for patients with biopsy-proven NASH, no specific treatment is recommended for this disease [7]. Consequently, in the absence of the effective therapy the lifestyle modification including low caloric diet, exercise, and weight loss are generally recommended treatments for patients with NAFLD [8, 9].

It is well recognized that epigenetic mechanisms of gene expression regulation can be influenced by the environmental factors such as diet or exercise, and disruption of those mechanism leads to the disease.

Enzymatic addition of methyl group to cytosines in DNA strand is referred to as DNA methylation. It is one of the major epigenetic mechanisms of gene expression regulation that in humans takes place almost exclusively within CpG dinucleotide. In general terms, when cytosines in the gene promoter are methylated, the gene is not expressed. This epigenetic mechanism of gene expression regulation is the most researched in the context of diet and methylation of various genes that have already been shown to be affected not only by specific nutrients such as dietary folate [10] or keto bodies [11] but also, more importantly, by diets including Mediterranean diet or Make Better Choices 2 (MBC2) [12–18]. Moreover, it becomes increasingly clear that apart from direct influence of the nutrition on methylome of the cells, the balance of the gut microbiota which is primarily regulated by diet plays a significant role in the nutritional status of the body and thus can influence epigenetic mechanisms of gene expression regulation [19].

As liver cells are affected in NAFLD, the investigation of casual effects of this disease should be performed using liver biopsies from the patients. We were not able to obtain liver biopsies in our study; however, previous research has already indicated that diet-induced epigenetic changes in blood and blood cells can to some extent be considered a surrogate of the effector cells [20–24]. Thus, we performed genome-wide methylation analyses of blood cells of NAFLD patients at the baseline and after 30 and 60 days from significant diet change. We then used publicly available data to investigate whether similar methylation changes occur in liver cells of NAFLD patients. Overall, we observed a pronounced effect of the dietary intervention on the methylome of blood cells. Moreover, we showed that methylation levels at loci identified to undergoing methylation change in blood cells during our study could also stratify liver biopsies according to the hepatic fibrosis grade.

## Results

### Participants adhered to the diet during study period

The median value of MDQI at inclusion was 5.5, clearly indicating that participants in general did not follow the Mediterranean diet. After 30 days (second timepoint), the index reached a median value of 7 indicating a significant improvement in the diet and at 60 days (third timepoint) the median index reached 8, which reflects close to ideal compliance with the Mediterranean diet (*p* value < 0.05) (Table 1 part A). Apart from the improvement in the MDQI, analysis of the components of the patients' diet showed that participants reduced the total daily fat as well as the total intake of carbohydrates and cholesterol (*p* value < 0.05) (Table 1 part B), increased the consumption of fibre (*p* value < 0.05), as well as food reach in methyl donors and cofactors involved in the metabolism of DNA methylation including folates, methionine, magnesium, vitamin B12, and riboflavin (vitamin B2) (*p* value < 0.05) (Table 1 part B).

**Table 1:** Characteristics of participants

Parameter (unit)	T1 median (range)	T2 median (range)	T3 median (range)	*p* val	*p* val T1 versus T2	adj. *p* val T1 versus T2	*p* val T1 versus T3	adj. *p* val T1 versus T3	*p* val T2 versus T3	adj. *p* val T2 versus T3
Age (year)	46 (29–68)									
*A. Adherence to diet*										
The Mediterranean Diet Quality Index	5.5 (1–9)	7 (5–9)	8 (4–10)	0.001	0.006	0.019	0.003	0.008	0.100	0.299
*B. Elements supplied with the diet (based on FFQ and 24-h dietary diary)*										
Energy (kcal/day)	2096.6 (1565–2851)	2018.79 (1650.1–2338.2)	1926.59 (1641.6–2413)	0.084	–	–	–	–	–	–
Fat (g/day)	97.11 (59–173.57)	56.52 (38.7–87.8)	55.9 (31.6–104.6)	8.63E−04	7.97E−04	0.002	0.001	0.003	0.744	1
Carbohydrates (g/day)	221.19 (177.5–378.37)	167.2 (95–280.9)	155.27 (91.8–283.8)	2.63E−05	0.003	0.010	1.62E−04	4.85E−04	0.090	0.269
Cholesterol (mg/day)	368.78 (122.3–641.8)	323.17 (181–617.1)	257.8 (140.1–541.5)	0.015	0.360	1	0.041	0.122	0.029	0.088
Protein (g/day)	74.82 (58.23–120.36)	81 (50.3–113.6)	77.1 (57.1–101.7)	0.667	–	–	–	–	–	–
Fibre (g/day)	16.54 (8.6–33.48)	32.44 (16–50.3)	32.57 (21.2–56.8)	1.74E−08	2.74E−06	8.22E−06	4.71E−06	1.41E−05	0.739	1
Folates (µg/day)	295.27 (146.3–580.82)	486.84 (295.5–750)	369.75 (192.9–794.7)	0.001	0.004	0.012	0.021	0.063	0.037	0.110
Methionine (mg/day)	1480.76 (1343–1758.5)	2031.59 (1319.8–2744.8)	1928.07 (1360.6–2793.1)	5.99E−05	7.97E−04	0.002	3.00E−04	9.01E−04	0.433	1
Magnesium (mg/day)	233.01 (109.4–310.7)	290.11 (188.2–395.2)	277.47 (146.3–461.1)	0.012	0.006	0.018	0.061	0.184	0.401	1
Vitamin B12 (mg/day)	3.41 (1.11–7.2)	6.7 (2.5–31.5)	3.75 (1.6–14.3)	0.016	0.006	0.018	0.257	0.772	0.010	0.031
Vitamin B6 (mg/day)	2.34 (0.9–6.6)	2.15 (1.4–2.8)	2.01 (1–3.2)	0.424	–	–	–	–	–	–
Vitamin C (mg/day)	121.77 (13.7–313.9)	114.7 (47–297.6)	116.72 (17–377.2)	0.678	–	–	–	–	–	–
Niacin (vitamin B3) (mg/day)	17.51 (8.38–39.57)	21.01 (11.2–28.6)	18.91 (6.4–32.2)	0.244	–	–	–	–	–	–
Riboflavin (vitamin B2) (mg/day)	1.4 (0.76–2.5)	1.86 (1–2.9)	1.3 (0.9–2.6)	0.004	0.016	0.047	1	1	0.049	0.148
Zinc (mg/day)	7.94 (4.06–13.43)	8.31 (4.6–10.5)	7.59 (5.7–10.9)	0.655	–	–	–	–	–	–
*C. Body parameters (Anthropometric)*										
Body weight (kg)	88.25 (62.9–106.4)	87.2 (61.9–104.9)	86.75 (62–103.8)	0.030	0.027	0.081	0.072	0.217	0.637	1
BMI (kg/m2)	30.63 (22.25–35.69)	29.66 (22.19–34.45)	29.16 (22.19–34.64)	0.033	0.029	0.088	0.075	0.226	0.641	1
Fat mass (%)	30.52 (18.5–43.8)	28.65 (18.6–43.3)	27.2 (17.7–43.6)	0.589	–	–	–	–	–	–
Muscle mass (kg)	54.7 (42.1–76.7)	53.4 (45.1–73.9)	53.55 (46.7–76)	0.358	–	–	–	–	–	–
Total body water (%)	50.55 (41.9–58.7)	51.25 (42.2–58.3)	51.3 (42–63.2)	0.242	–	–	–	–	–	–
*D. Liver depending biochemical parameters analysed in blood*										
ALT (U/I)	43.5 (11–136)	41.5 (9–95)	38 (11–86)	0.656	–	–	–	–	–	–
AST (U/I)	28 (11–52)	26.5 (10–59)	27.5 (13–40)	0.500	–	–	–	–	–	–
GGTP (U/L)	28.5 (12–114)	23.5 (12–98)	23.5 (11–118)	0.013	0.003	0.010	0.433	1	0.023	0.069
Total cholesterol (mg/dl)	207.45 (146.9–394.4)	181.6 (109–330.5)	189.1 (103.9–340.2)	0.006	4.20E−04	0.001	0.010	0.031	0.027	0.081
LDL (mg/dl)	139.55 (87.6–282.2)	113 (62.4–251.2)	124.9 (58.9–258.3)	0.005	0.002	0.006	0.060	0.180	0.014	0.042
HDL (mg/dl)	47.5 (32.8–67.1)	44.7 (32.2–62.9)	45.9 (32.5–63.7)	0.155	–	–	–	–	–	–
HDL (mg/dl) Female (*n* = 10)	46.6 (32.8–67.1)	45.45 (32.2–62.9)	43.6 (32.5–63.7)	0.69	–	–	–	–	–	–
HDL (mg/dl) Male (*n* = 8)	48.25 (40.9–52.3)	44.7 (38.9–51.5)	47.4 (41.6–51.6)	0.04	0.065	0.195	0.97	1	0.02	0.065
Glucose (mg/dL)	91.2 (79.6–174.5)	94.1 (83.9–143.3)	91.55 (76.3–125.2)	0.546	–	–	–	–	–	–
Insulin (mU/ml)	18.55 (6.7–152)	23.05 (4.2–91.8)	18.4 (4.3–86.7)	0.412	–	–	–	–	–	–
Triglycerides (mg/dl)	168.9 (76.1–517.6)	153.7 (68.1–432.8)	155.1 (73.1–334.7)	0.206	–	–	–	–	–	–
*E. SCFAs in stool*										
Acetic acid (mmol/l)	2.25 (0.87–5.1)	2.72 (2.18–8)	3.55 (2.06–4.94)	0.047	0.148	0.444	0.010	0.031	0.496	1
Propionic acid (mmol/l)	0.72 (0.31–2.12)	0.79 (0.29–2.4)	1.09 (0.6–1.61)	0.161	–	–	–	–	–	–
Isobutyric acid (mmol/l)	0.02 (0–0.17)	0.01 (0–0.11)	0.05 (0–0.28)	0.104	–	–	–	–	–	–
Butyric acid (mmol/l)	0.48 (0.12–2.07)	0.55 (0.3–3.55)	1.09 (0.25–1.95)	0.002	0.468	1	0.007	0.020	0.009	0.027
Isovaleric acid (mmol/l)	0.01 (0–0.19)	0.01 (0–0.15)	0.04 (0–0.3)	0.349	–	–	–	–	–	–
Valeric acid (mmol/l)	0 (0–0.13)	0 (0–0.3)	0 (0–0.25)	0.231	–	–	–	–	–	–
*F. SCFAs in plasma*										
Acetic acid (µM/L)	26.51 (10.38–52.33)	21.13 (8.76–48.97)	23.35 (10.74–52.14)	0.464	–	–	–	–	–	–
Propionic acid (µM/L)	1.1 (0.41–2.1)	0.76 (0.4–3.73)	0.81 (0.53–2.38)	0.846	–	–	–	–	–	–
Isobutyric acid (µM/L)	0.11 (0–0.19)	0.08 (0–0.18)	0.09 (0.05–0.19)	0.132	–	–	–	–	–	–
Butyric acid (µM/L)	0.39 (0.14–1.67)	0.4 (0.12–2.11)	0.41 (0.09–2.01)	0.223	–	–	–	–	–	–
2-Methylbutyric acid (µM/L)	0.11 (0–0.53)	0 (0–0.29)	0 (0–0.1)	0.015	0.011	0.033	0.009	0.027	0.845	1
Isovaleric acid (µM/L)	0.49 (0–1.53)	0.24 (0.1–0.82)	0.29 (0–0.7)	0.069	–	–	–	–	–	–
Valeric acid (µM/L)	0 (0–0.28)	0 (0–0.19)	0 (0–0.18)	0.009	0.151	0.452	0.022	0.067	1	1
Isocaproic acid (µM/L)	0.08 (0–0.26)	0 (0–0.13)	0 (0–0.15)	0.014	0.009	0.026	0.011	0.032	0.675	1
Caproic acid (µM/L)	0.47 (0.33–1.18)	0.44 (0.24–0.78)	0.52 (0.34–0.83)	0.234	–	–	–	–	–	–
*G. Liver steatosis and stiffness status*										
Liver steatosis–CAP (dB/m)	310 (242–400)	–	279.5 (228–371)	0.035	–	–	–	–	–	–
Liver stiffness–VCTE (kPa)	6.05 (3.9–9.4)	–	5.4 (4–9.6)	0.200	–	–	–	–	–	–

^*^T1 Timepoint 1; *T2 *Timepoint 2; *T3 *Timepoint 3; *p val* *p* value

### Effect of the diet on anthropometric, biochemical, liver steatosis, stiffness status and SCFAs of participants

Considering changes in the indicators of the compliance with dietary recommendation during the study, not surprisingly, we observed a statistically significant decrease in weight and BMI (*p* value < 0.05), which at inclusion indicated obesity. However, the decrease was rather minor and the change in other parameters did not reach statistical significance (Table 1 part C). Furthermore, we observed an improvement in the key biochemical parameters (Table 1 part D) including GGTP, total cholesterol and LDL cholesterol (*p* value < 0.05). We also analysed the concentration of SCFAs in stool and blood of study participants. The SCFAs are the products of gut bacterial anaerobic fermentation of dietary fibre and likely reflect an improvement in the gut microbiome. Although concentration of all analysed SCFAs in stool increased, the increase reached statistical significance for only acetic acid and butyric acid (*p* value < 0.05) (Table 1 part E). The SCFAs levels in plasma generally decreased during the study, and the changes in levels of three of them, including 2-methylbutyric, valeric and isocaproic acids, were statistically significant (*p* value < 0.05) (Table 1 part F). But there was no statistically significant correlation between levels of SCFAs (measured in blood or stool) and hepatic steatosis status. The most interesting observation in this part of the study was the statistically significant decrease in liver steatosis (Table 1 part G) from a baseline of 310 dB/m, characteristic for medium- to high-grade steatosis to 279.5 dB/m at the third timepoint (*p* value < 0.05). The median liver stiffness of participants was also reduced, but that change was not statistically significant (Table 1 part G).

### Change in the dietary habits appears to reduce acetylation levels of lymphocytes and monocytes

**Fig. 1:**
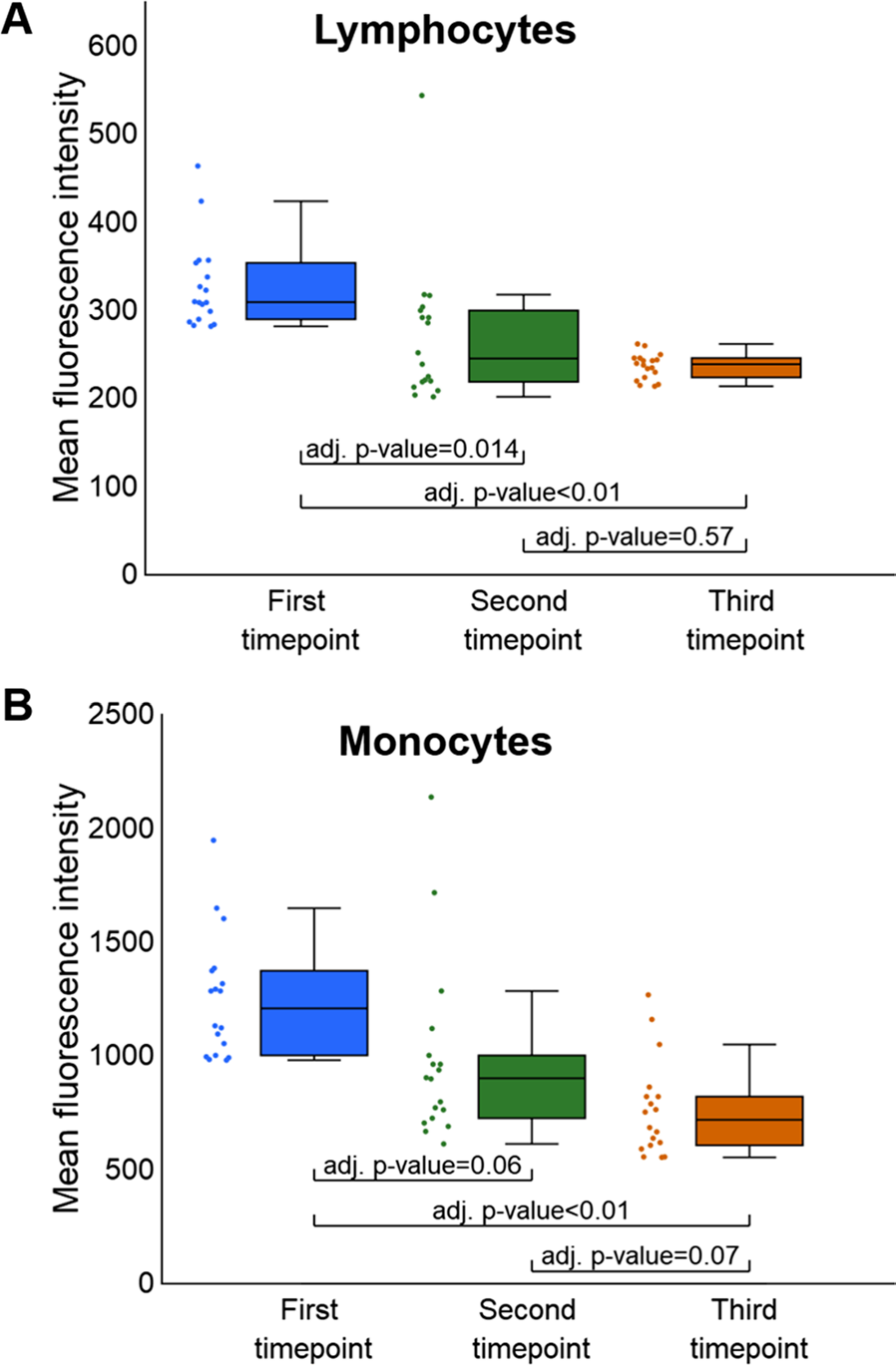
Analysis of relative histone acetylation changes in lymphocytes (**A**) and monocytes (**B**) between study timepoints. **A** The change in the fluorescence levels for lymphocytes was statistically significant between the first and both the second and the third timepoints. **B** In monocytes, we observed a statistically significant decrease in detected fluorescence between the first and the third timepoints. The change was not statistically significant between the first and second timepoints. We did not observe statistically significant differences in the levels of fluorescence intensity between the second and third timepoints in neither lymphocytes (**A**) nor monocytes (**B**)

With the indication that the intervention in our study not only improved the anthropometric, biochemical and liver steatosis status, we investigated the relative change in the histone acetylation of the lymphocytes and monocytes. This change would suggest a potential effect of intervention on epigenome. The peripheral blood mononuclear cells were stained with antibodies specific to acetylated histone H3, and we used the flow cytometry to measure the fluorescence intensity of stained lymphocytes and monocytes. The change in the fluorescence levels for lymphocytes was statistically significant between the first and both the second and the third timepoints (*p* value < 0.05) (Fig. 1A). For monocytes, we observed a statistically significant decrease between the first and the third timepoints (*p* value < 0.05) (Fig. 1B). At the same time, the change in the fluorescence was not statistically significant between the second and third timepoints (Fig. 1A, B). Deacetylation of histone H3 on lysines 9 and 14 has long been associated with actively transcribed loci [25, 26] and inversely correlated with the DNA methylation [27]; therefore, we went on to assess the influence of diet modification on methylome of blood cells.

### The diet change influences methylation pattern of blood cells

**Fig. 2:**
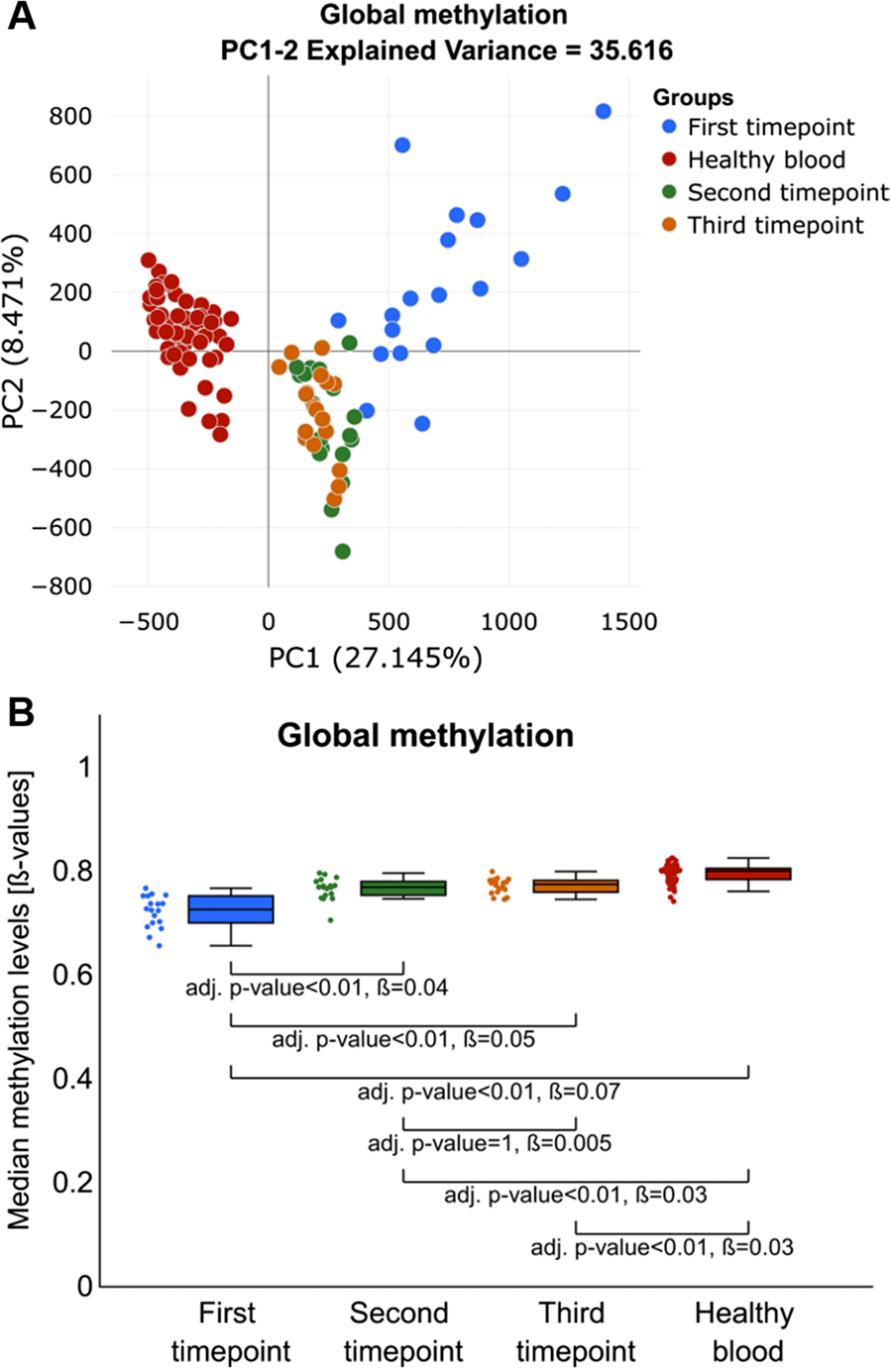
Analyses of global methylation changes between study timepoints. **A** PCA plot indicating that genome-wide methylation of blood profiles of participants before intervention (blue colour) are different and more heterogeneous than profiles from the second timepoint (green colour), third timepoint (orange colour) and healthy controls (red colour). **B** Comparison of median global methylation levels indicates an increase in methylation between the first and both the second and the third timepoints of the study with global methylation levels at the second and third timepoint resembling the methylation level of healthy blood (red colour)

To assess the influence of the diet change on genome-wide methylation patterns of the blood cells, we first compared the methylation profiles between study timepoints. Additionally, we used methylation profiles of blood from healthy controls as a reference in that comparison. Already initial principal component analysis (PCA) based on combined methylation changes at 666,589 informative CpGs obtained for each of the participants in the study showed that methylation profiles from the second and third study timepoints were very similar and grouped together (green and orange colours for second and third timepoints in Fig. 2A, respectively). Those profiles also clearly represented intermediate methylation between the first study timepoint (marked in blue in Fig. 2A) and healthy blood controls (marked in red in Fig. 2A), indicating that the change in genome-wide methylation patterns during the study was towards the pattern characteristic for healthy blood. We then calculated and compared median global methylation levels between study timepoints and healthy blood controls. Also in this analysis, the most pronounced change in the median global methylation was between the first and second/third timepoints (Fig. 2B), whereas the median global methylation levels at the second and the third timepoints were intermediate between the first timepoint and the healthy blood.

We also tested whether observed in PCA plots heterogeneity between methylation profiles at the first study timepoint is attributed to any of the physiological parameters we recorded for the participants or proportions of the cell fractions in the samples. Pearson correlation between each of those parameters and the median global methylation levels was not statistically significant (adj. *p* value > 0.05) (Additional file 1: Table S1), and the proportions of the cells in blood samples at the first study timepoint were not statistically significantly different from the levels of those cells in healthy blood (*p* value > 0.05) (Additional file 1: Fig. S1).

### Diet change affects methylation levels only at a specific subset of CpG sites

**Fig. 3:**
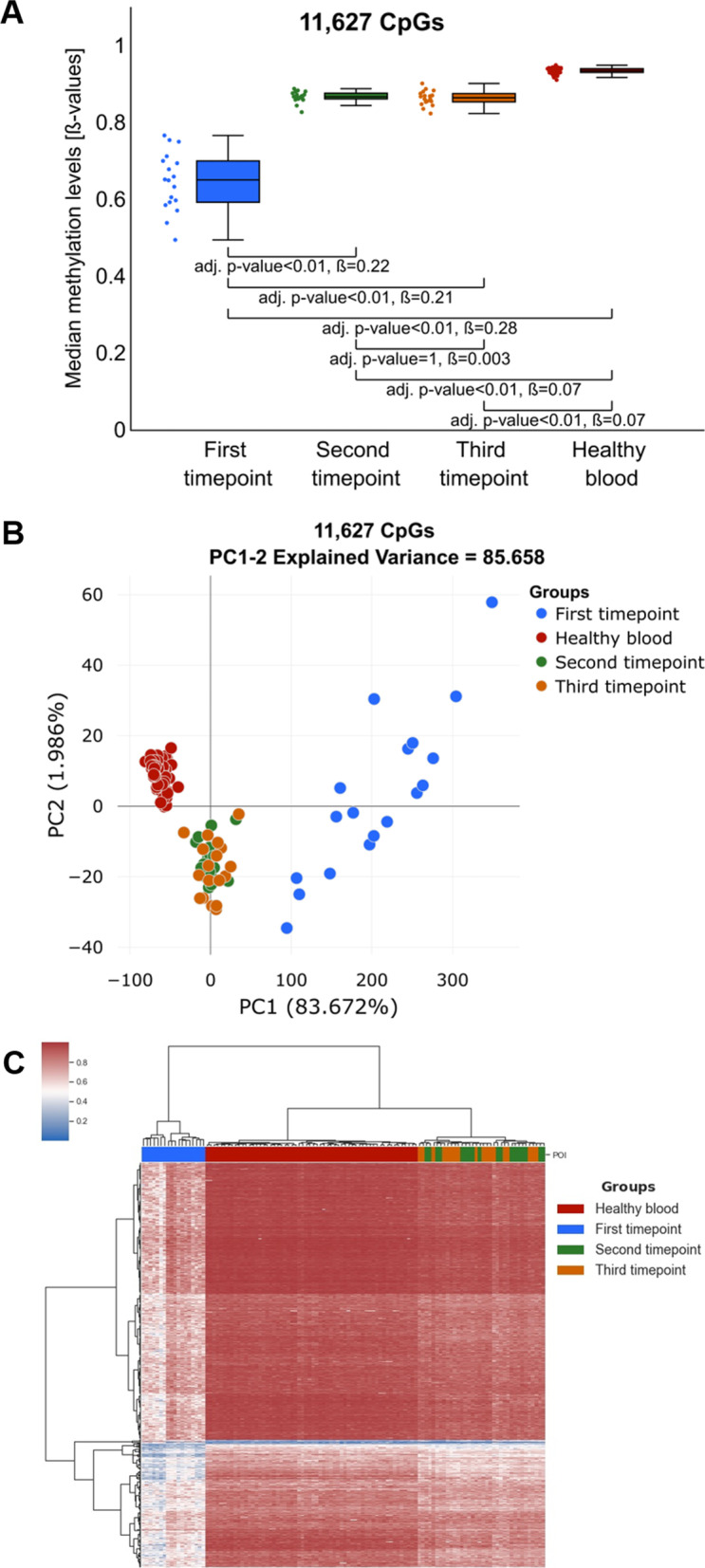
Comparison of the methylation changes at a subset of CpG sites that we identify to display diet-related methylation changes. **A** Comparison of the median methylation levels for analysed groups including: first (blue), second (green), third (orange) timepoints and healthy controls (red). The difference at identified CpGs is significantly larger than the ones observed in Fig. 3B, indicating that methylation at this subset of CpG sites was most affected in the study. **B** PCA plot based on the identified subset of CpG sites indicates a significant change in the methylation profile of this subset of CpG sites between the first and the second/third timepoints. **C** Heatmap illustrating unsupervised clustering analyses indicates significant hypomethylation (blue colour in the heatmap) at the first study timepoint and significant increase in the methylation level towards the levels observed in healthy controls (red colour in the heatmap) at the second/third timepoints

Then, to identify CpG sites most affected by the diet change, we compared the methylation levels at each of the informative CpGs, between the first and the average methylation level at the second and third study timepoints. The detailed description of the analysis is in Additional file 1: Methods 1, and for the result of validation of this method see Additional file 1: Fig. S2. This analysis resulted in 11,627 deferentially methylated CpG sites with more than 10% methylation difference. As expected, the majority of the observed methylation changes were gains of methylation with 11,485 CpG sites displaying hypermethylation and only 142 hypomethylation (for the examples of methylation changes at specific CpGs see: Additional file 1: Fig. S3). The median methylation levels at this subset of CpG sites at the second and the third timepoints of the study were again intermediate between the first study timepoint and healthy blood, but very similar to the methylation levels in healthy blood (Fig. 3A). Interestingly, observed methylation changes were larger than median global methylation changes, indicating that these CpG sites were indeed most affected by the diet change. Also, the variance of the methylation levels at these CpG sites was statistically significantly higher at the first timepoint (var = 0.0058) than at all other timepoints and healthy blood (second timepoint var = 0.0002, third timepoint var = 0.00036 and healthy blood var = 0.00005; *p* value < 0.05). Again, here we tested the association of the average methylation levels at this subset of CpGs sites with the biochemical parameters recorded for patients at this timepoint and found no statistically significant associations (Additional file 1: Table S2).

We then performed PCA and unsupervised hierarchical clustering analysis of methylation levels at this subset of CpG sites. The first component in this PCA (Fig. 3B) explained more than 83% of the variance confirming that the global methylation changes that we observed in previous analysis were mainly driven by the methylation changes at this subset of CpG sites. Moreover, both PCA and unsupervised clustering showed that the direction of the diet-induced methylation changes at the second and the third timepoints (marked with green and orange in Fig. 3B, C, respectively) was clearly towards methylation changes observed in healthy blood samples (marked with red in Fig. 3B, C).

### Diet-related methylation changes are enriched in region not directly involved in gene expression regulation

We then analysed whether identified CpG sites are enriched in specific functional genomic regions such as gene elements, CpG islands and gene regulatory regions (Additional file 1: Fig. S4). This analysis showed that the identified CpG sites were enriched in the regions not directly involved in gene expression regulation including Exon boundary (FC = 1.45), Gene Body (FC = 1.27), 3’UTR (FC = 1.83), Open Sea (FC = 1.52), North Shelf (FC = 1.5), South Shelf (FC = 1.48), Enhancer (FC = 1.65) and Open chromatin region (FC = 1.47) and depleted in regions considered to directly control gene expression including TSS1500 (FC = 0.7), TSS200 (FC = 0.2), 5’UTR (FC = 0.8), 1st Exon (FC = 0.2), CGIs (FC = 0.03), North Shore (FC = 0.46) and South Shore (FC = 0.45), Promoters (FC = 0.11) and CTCF binding site (FC = 0.53). At the same time, the frequency of the CpG sites with intervention-related methylation changes was almost the same as expected by chance in Promoter flanking region (FC = 1.04) and TF binding site (FC = 1.1). The identified CpG sites also did not localize to specific chromosomes and were not significantly enriched in histone binding sites (Additional file 1: Table S3) and in specific transcription factor bounding sites (Additional file 1: Table S4).

### Functional annotation of the diet-related methylation changes

The previous analysis indicated that the majority of the identified 11,627 CpG sites displaying methylation changes in our study despite that they mapped to 9956 genes (listed in Additional file 2) were not located in the regions traditionally associated with gene expression regulation (e.g. promoters). Recently, however, the regulatory function of the methylation changes outside of traditional regulatory regions is reported in increasing number of publications [28–30]. Therefore, we mapped the identified CpGs to the gene regions and used FUMA pipeline for functional mapping and annotation of obtained gene set [31]. This analysis showed that liver was one of four tissue types where identified genes were shown to undergo downregulation. Other tissue types identified in this analysis were “Kidney_Cortex”, “Artery_Tibial” and “Nerve_Tibial”. Interestingly, we were able to find publications linking all of those tissues types to NAFLD (Additional file 1: Table S5A). We then explored the functional annotation of genes in this gene set in nine gene term databases (listed in Additional file 1: Table S5B) and interrogated Google Scholar for the publications reporting result associating NAFLD with top hit terms in each of the databases. Surprisingly, we were able to reference more than 70% of terms identified in each of the databases to the publications describing physiological processes involved in pathology of the NAFLD. The details of this analysis and references to the identified literature are listed in Additional file 1: Table S5.

### Methylation changes observed in blood separate liver biopsies from NAFLD patients according to the fibrosis grade

**Fig. 4:**
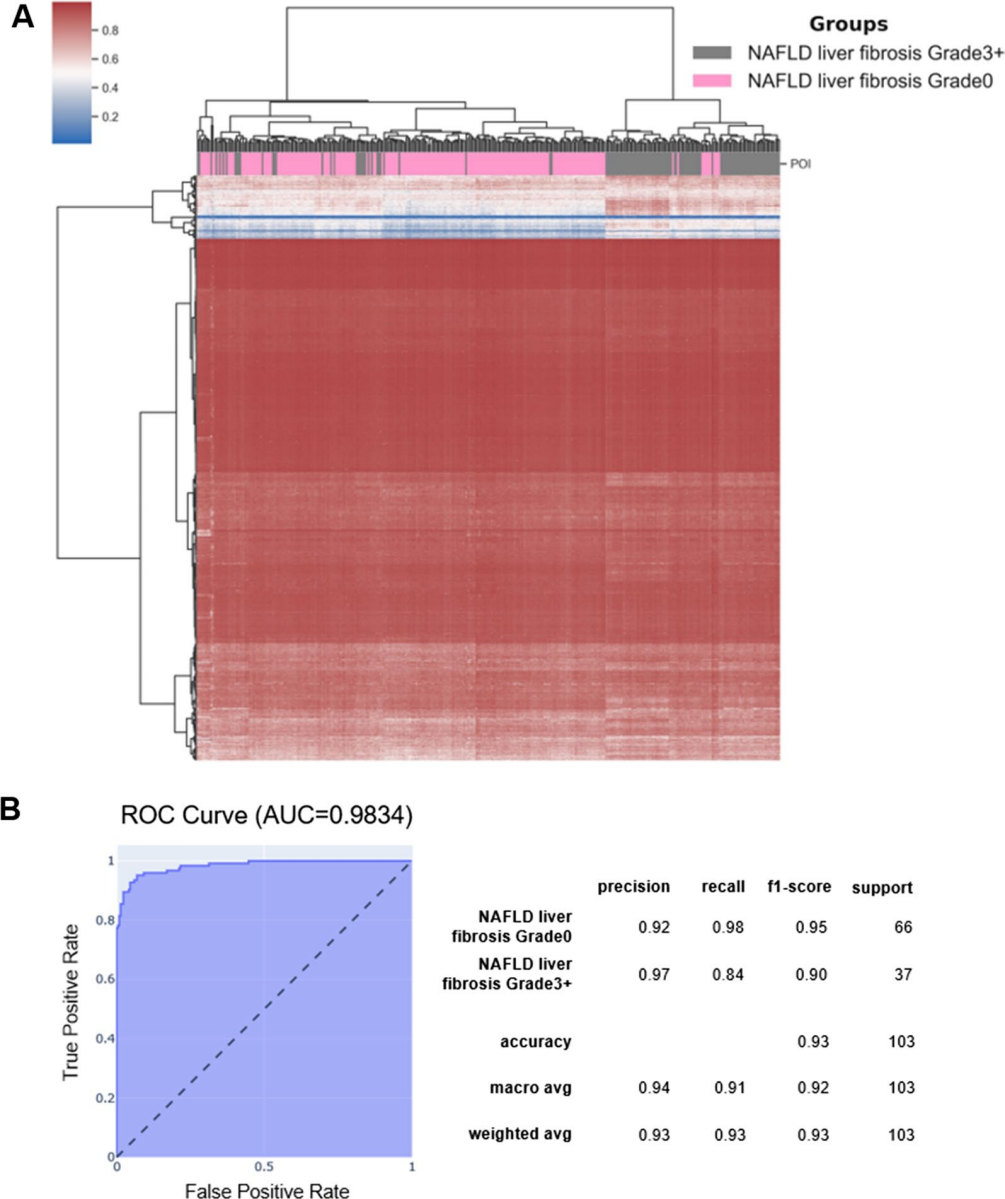
Classification of the NAFLD biopsies. **A** Heatmap representing unsupervised clustering of the biopsies and the fibrosis grade assessed by the pathologist. **B** ROC curve describing the accuracy of the ML-based classification of NAFLD patients

As already mentioned, the major limitation of studies like ours is the inability to obtain liver tissue from the participants, which would allow identification of the methylation changes directly implicated in NAFLD. There is, however, evidence that at least to some extent the methylation changes observed in blood cells can be considered a proxy of changes observed in effector tissue [20–22, 24]. In recently published paper, the association of the methylation changes in liver with the fibrosis progression was investigated [32]. We used data from this study that included methylation profiles of the 341 liver biopsies to validate to what extent observed in blood cells methylation changes reflect the methylation aberrations in liver cells of NAFLD patients. The unsupervised clustering of liver biopsies based on methylation changes at the subset of CpG sites was able to remarkably accurately separate liver biopsies according to the grade of the fibrosis with *p* value = 2.62e−38 (Chi^2^) (Fig. 4). Moreover, we used tree-based automated machine learning (ML) [33] to assess the accuracy of classification of grade 0 and grade 3 + fibrosis based on this subset of CpG sites (for details of ML analysis see Additional file 1: Methods 2). With the ML approach, we were able to distinguish grade 0 and grade 3 + fibrosis with the accuracy of 0.93 and the ROC from this analysis had AUC = 0.9834 (Fig. 4B).

### Methylation changes in blood as a biomarker of the methylation changes in liver

**Fig. 5:**
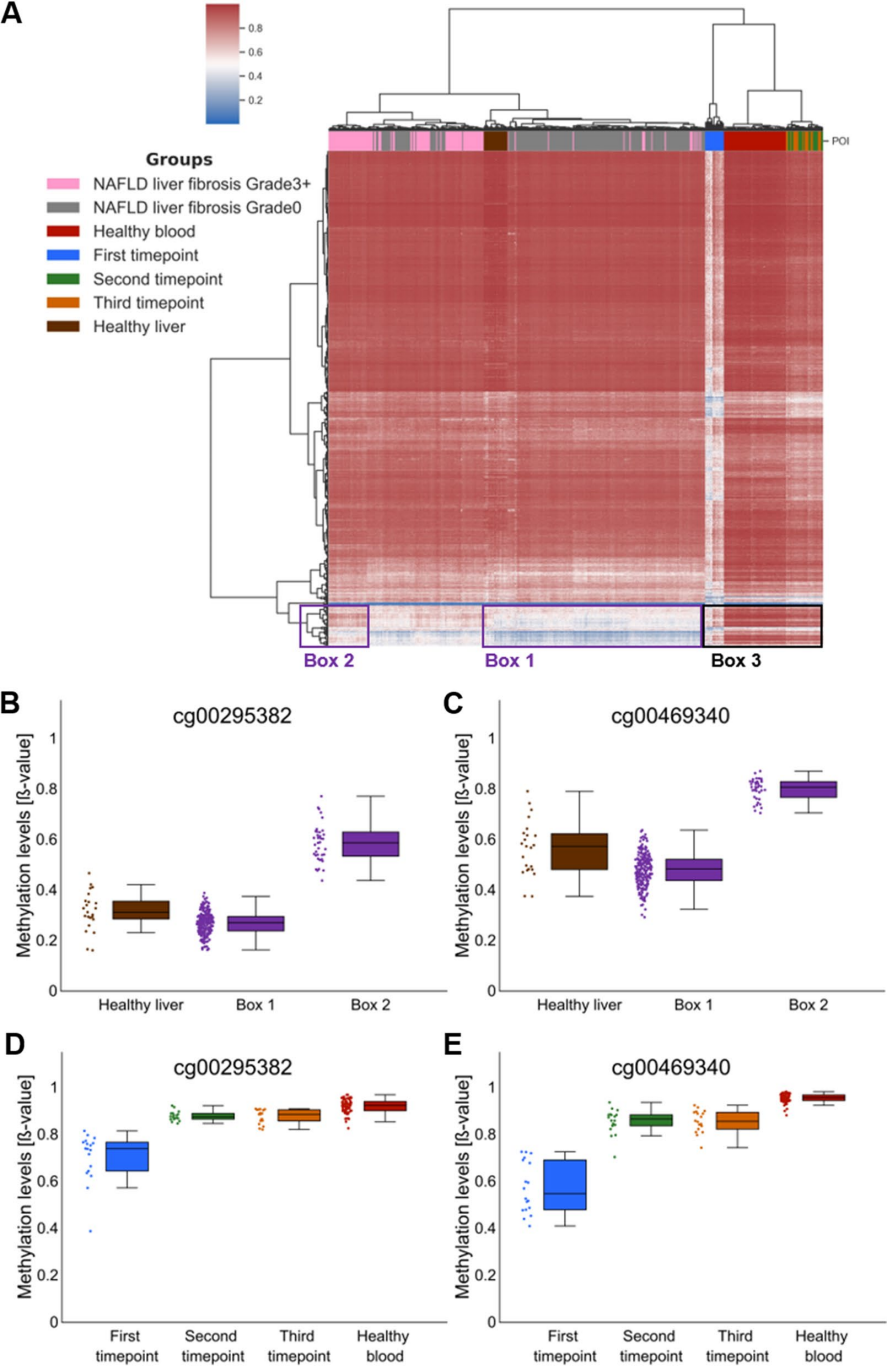
Comparison of the methylation levels at the subset of the CpG sites identified in our study between our study timepoints, healthy blood (GSE123914), healthy livers (GSE136380) and the liver biopsies of NAFLD patients (GSE180474). **A** Unsupervised clustering that includes liver biopsies and our study participants. **B–E** Box plots with the examples of the tissue-specific methylation changes

We also used unsupervised clustering to compare the methylation levels at the identified subset of CpGs between liver biopsies, healthy blood, healthy liver and blood from all the timepoints in our study. This analysis showed that the methylation levels at those CpGs were very similar between healthy blood (Fig. 5A—red colour) and liver (Fig. 5A—brown colour) but significantly different from blood of our participants before diet change (Fig. 5A—blue colour). At the same time, the methylation levels at the second and the third timepoint of our study (Fig. 5A—green and orange colours) were very similar (clustered together) to healthy blood. This suggests a potential use of the methylation changes at these CpGs as blood-based biomarkers of the NAFLD. We also compared the methylation levels at the single CpG site between all groups of patients in the study. The representative results from this comparison are shown in Fig. 5B, C and corroborate the results of the heatmap.

### Fibrosis-specific methylation changes

Interestingly, the unsupervised clustering analyses depicted in Fig. 5 showed that a subset of CpG sites displays very similar methylation levels in healthy liver (brown colour) and Grade 0 biopsies (grey colour) (Fig. 5A—Box 1, Fig. 5B, C). At the same time, those CpG sites display specific methylation changes in biopsies with grade 3 fibrosis (pink colour) (Fig. 5—Box 2, Fig. 5B, C). This suggests that methylation at this subset of CpG sites is tissue-specific and the progression of fibrosis not surprisingly involves changes of tissue-specific methylation patterns. We also compared the methylation levels at these CpG sites in blood between timepoints of our study (Fig. 5A—Box 3, Fig. 5D, E). This analysis again showed that methylation of this subset of CpG sites was also altered by the diet change in blood cells and can be a potential biomarker of the fibrosis progression. However, as our study did not include analysis of the methylation of blood from patients with grade 0 and 3 + fibrosis, this needs further investigation.

## Discussion

Diet has been shown to have a pronounced effect on the methylomes of blood cells [10–18]; for example, LINE-1 elements of blood cells not only differ between cases and controls following and not following the diet, but more importantly between groups reporting different levels of adherence to the diet [34]. Moreover, recently patients with NASH have been shown to exhibit epigenetic age acceleration measured by methylation changes in blood [35].

We studied genome-wide methylation changes in blood from individuals with NAFLD who changed the dietary habits and followed Mediterranean diet for 60 days that resulted in a statistically significant improvement in the liver function measured by liver steatosis.

Our analysis identified a subset of 11,627 CpG sites at which the methylation levels were significantly altered after diet change. This indicates a pronounced effect of the dietary intervention on the blood cells’ methylome of individuals with NAFLD. The most interesting, however, was finding that the identified methylation changes did not include gene regulatory regions such as transcription start sites (TSS) but span gene body, exon boundary and 3’UTR. The presence of methylation in gene body has been shown to increase the fidelity and efficiency of gene transcription by confining the transcription to the canonical promoters [36, 37]. Moreover, it has been shown in cancer that intragenic methylation can alter expression of at least some genes [38]. Our results therefore suggest that the change in the diet in our study may have improved gene transcription; more importantly, gene set enrichment analysis confirmed the involvement of the genes annotated to the identified methylation changes in the physiology of the NAFLD and we were able to find literature references associating more than 70% of the ontology terms from these analyses with NAFLD.

One drawback of studies similar to ours is the inability to obtain liver tissue from the participants due to the risks related to the liver biopsy procedure. We have, however, been able to analyse the methylation at the identified subset of CpG sites in liver biopsies from previous study. This analysis showed that to a large extent the methylation changes in blood reflect methylation changes in the liver of NAFLD patients. Moreover, we have shown that we are able to, with remarkable accuracy, classify the grade of liver fibrosis in biopsies on the bases of the methylation changes at the CpG sites that we identify to undergo diet-related methylation changes in blood. That shows that at least to some extent we can consider methylation changes in blood, a proxy of the changes that occur in liver.

The results of the methylation profiling in our study are in line with the results of absolute measurements of histone acetylation in lymphocytes and monocytes of our study participants, where we observed a decrease in the relative acetylation. As already mentioned, DNA methylation and acetylation interplay in gene transcription regulation and deacetylation of histone H3 on lysines 9 and 14 has long been associated with actively transcribed loci [25, 26] and inversely correlated with the DNA methylation [27].

The specific molecular mechanisms by which diet influences methylation and acetylation of the tissues are not known, and the results of our study only identify a pronounced effect of diet on those processes in blood cells. Undoubtedly, the level of supplementation of the donors necessary in both histone acetylation and DNA methylation in the diet will have an impact on the metabolism of those two processes. The animal studies have repeatedly shown that supplementation or reduction in dietary methyl donors modifies DNA methylation levels in liver cells as well as results in liver fat changes [39, 40]. Nevertheless, further studies are needed to elucidate molecular mechanisms of interaction between diet and epigenome of specific tissues.

One intriguing finding in our study is the observed increase in short-chain fatty acids (SCFAs) in stools of participants after diet change. The primary source of the SCFAs is gut microbiome [41] that suggests that the diet in our study affected and improved the gut microbiome of the participants. Moreover, SCFAs have been shown to affect the activity of histone acetylase and histone deacetylase enzymes that would provide an elegant mechanism for the interaction, but this hypothesis needs further investigation.

One limitation of our study is lack of healthy controls following Mediterranean diet, and we only compare methylomes of NAFLD patients before and after intervention to the controls for which we were not able to access the dietary information. In the following study, adequate controls group should be used; nevertheless, the compression that we were able to make clearly indicates the effects of the intervention.

## Conclusions

In conclusion, our results clearly indicate a pronounced effect of the diet on the epigenetic mechanism of gene expression regulation and link those changes to the aetiology of the NAFLD.

## Materials and methods

### Study design

**Fig. 6:**
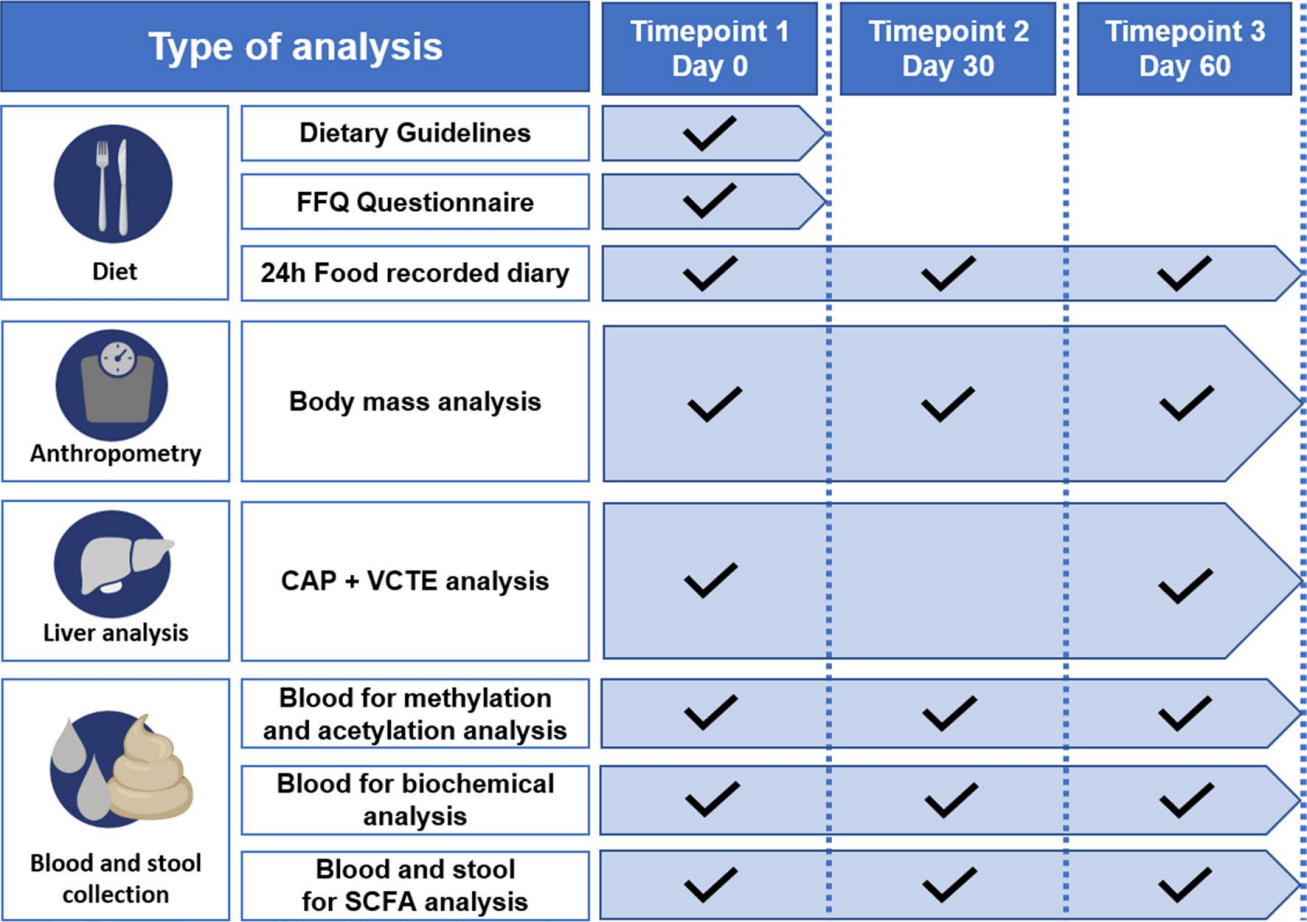
Study design and outline of analyses performed at each timepoint

The study was conducted between July and November 2019. Participants were recruited at Sonomed Medical Centre in Szczecin, Poland. After inclusion, participants undergone three rounds of clinical assessments at day 0 (baseline/first timepoint), after 30 days (second timepoint) and 60 days (third timepoint). At each timepoint, specific clinical parameters were recorded and clinical material was collected as illustrated in Fig. 6. Overall, the group of 18 eligible participants that included Caucasian men (*n* = 8) and women (*n* = 10) with NAFLD were recruited following the criteria: FibroScan^®^ (CAP ≥ 234 dB/m), age > 18 years old and no diagnosis of inflammatory gut disease. We used Food Frequency Questionnaire (FFQ) [42] (detailed description in Additional file 1: Methods 3) to assess dietary habits of the participants for a 6-month period before enrolment with daily intake of the nutrients calculated using nutrient analysis software Dieta5, version 5.8.2. (Warsaw, Poland) with the reference to the database of the Polish National Institute of Nutrition (https://www.pzh.gov.pl/wp-content/uploads/2020/12/Normy_zywienia_2020web-1.pdf). The participants included in the study largely did not comply with the Mediterranean diet as shown by the Mediterranean Diet Quality Index (MDQI) score (detailed description in Additional file 1: Methods 4) (Table 1 part A) [43]. We also excluded participants that at enrolment or during the study were confirmed with: infection with either HBV (hepatitis B virus) or HCV (hepatitis C virus), body mass index (BMI) > 35 kg/m^2^, displayed increased physical activity before the study or reported changes in physical activity during the study (detailed description of physical activity assessment in Additional file 1: Methods 5), reported the excessive consumption of alcohol (> 30 g in men and 20 g in women per day), excessive drug use or reported any condition that could limit the mobility of the participant. The study protocol was approved by the ethics committee of the Pomeranian Medical University (Szczecin, Poland, KB-0012/131/19) and conformed to the ethical guidelines of the 1975 Declaration of Helsinki. The volunteers provided written informed consent before the study.

### Guidelines and control of diet adherence

Each participant was then individually instructed by licensed nutritionist about principles of the Mediterranean diet and received pamphlet with detailed dietary recommendations to follow (Additional file 1: Methods 6 and Fig. S5). Additionally, participants were requested to eat two specifically formulated high-fibre rolls (buns) on each day during the study (Additional file 1: Methods 7). Twenty-four-hour food diaries were used to control participants adherence to diet (detailed description in Additional file 1: Methods 8). The changes in diet between timepoints were evaluated using MDQI scale [43]. Moreover, the participants were instructed not to exceed moderate activity (cardio training 5x/week every day, resistance training > 2x/week; no more than 150–300 min/week).

### The anthropometric data

Anthropometric assessments were performed routinely at each of the three timepoints of the study. The measurements included: height (m), body weight (kg) and body composition including: fat mass (%), muscle mass (kg) and total body water (%), which was assessed with the multifrequency bioimpedance meter, Tanita BC-601 (Japan).

### Liver stiffness and steatosis measurements

Liver stiffness (VCTE) and steatosis (CAP) were measured with FibroScan^®^ at baseline and at third timepoint by the same trained in technology MD (detailed description in Additional file 1: Methods 9).

### Short-chain fatty acids (SCFAs) analysis

Gas chromatography analyses of SCFAs in stool were performed according to procedure described in Additional file 1: Methods 10 and analyses of SCFAs in plasma were performed using Waters Acquity Ultra Performance Liquid Chromatography and Waters TQ-S triple quadrupole mass spectrometer as described in Additional file 1: Methods 11.

### Histone acetylation analysis

Acetylation of peripheral blood lymphocytes and monocytes was measured using Milli-MarkTM Anti-Acetyl-Histone H3-PE (Milli-Mark, Merck, Germany) and flow cytometry, following the manufacturer’s protocols (detailed description in Additional file 1: Methods 12).

### DNA extraction for methylation analysis

The DNA from the blood samples at the first timepoint was extracted using Invisorb^®^ Spin Blood Mini Kit (Stratec Biomedical AG, Germany) following the manufacturer’s instructions and salting out method from the second and third timepoints.

### Genome-wide methylation analysis

The genome-wide methylation changes screening in DNA extracted from whole blood was performed using Illumina MethylationEPIC BeadChip (EPIC, Illumina Inc.). The raw data were processed with ChAMP pipeline and normalized with BMIQ method. This data processing resulted in 666,589 informative CpGs available for all the participants at each timepoint of the study. Considering the data structure, we developed a specific data processing approach to identify the loci, at which methylation was most affected by the intervention (detailed description in Additional file 1: Methods 1). Briefly, the ∆β representing difference in methylation before and after the intervention was generated by subtracting β value for each CpG site at the first timepoint from averaged β value from the second and third timepoints, for each patient separately. Then, to take into account heterogeneity of the methylation profiles observed at the first timepoint we only included in further analyses CpGs that displayed 10% difference in methylation for more than 3/4 (77%) of the participants. Overall, this procedure resulted in 11,627 CpGs displaying differential methylation before and after intervention. EpiDISH R package [44] was used to assess and compare cell fraction proportion of the blood samples at the first timepoint and the group of healthy individuals from GSE123914 that we used as healthy controls in our study [45].

### Gene ontology term enrichment and fold change analyses

We performed gene set enrichment analysis using “GENE2FUNC” function in FUMA [46] to establish which biological pathways may be affected by the identified methylation changes. We also used HOMER v4.11 for motif discovery analysis [47] and ChIP-Atlas to predict multiple histone modifications at the loci where we identified methylation changes. To investigate whether identified methylation changes occupy specific genomic regions, we analysed the distribution of those loci relative to the CpG islands, gene regions feature category (according to the Infinium MethylationEPIC v1.0 B4 Manifest File, UCSC coordinates) and regulatory regions as defined in Ensembl [48].

### Statistical analysis

The change in the parameters recorded for each of the participants at all study timepoints was tested with repeated measures ANOVA test. If the data did not meet the conditions for parametric test, then the Friedman test was used. For the parameters with significant associations, the post hoc paired *t* test or Wilcoxon test was performed. For the comparison of the parameters between two timepoints, we used paired *t* test or Wilcoxon test. Independent *t* test or Mann–Whitney tests were used for comparison of parameters from each of the timepoints to healthy controls and Pearson test to calculate the correlation between median methylation levels and clinical parameters. All analyses were corrected for the multiple testing using Bonferroni correction. The missing data points were imputed with mean of the parameter. Statistical significance was declared at p-value less than 0.05. All analyses were performed in R 4.0.3 environment.  Chi-square test and TPOT tool was used for testing the classification accuracy of liver fibrosis grade based on identified methylation changes [33].

### Controls and validation data

Publicly available EPIC methylation profiles were used as healthy blood controls (GSE123914, *n* = 60) [45], and this group did not undergo the dietary intervention. To validate final results, we used GSE180474—NAFLD liver samples with defined fibrosis stage (*n* = 341) [32], and GSE136380—pathologically confirmed healthy livers (*n* = 23) [49].

## Supplementary Information

**Additional file 1.** Supplementary Methods and Results.

**Additional file 2.** 11627 CpGs annotated to genes.

## Data Availability

The microarray data generated in this study will be deposited in GEO and will be released upon publication. Accession numbers of the publicly available data used in this study are as follows: GSE123914, GSE180474, and GSE136380.

## Abbreviations

NAFLD: Non-alcoholic fatty liver disease
HCC: Hepatocellular carcinoma
LD: Lipid droplets
MBC2: Make better choices 2
FFQ: Food frequency questionnaire
MDQI: Mediterranean diet quality index
HBV: Hepatitis B virus
HCV: Hepatitis C virus
BMI: Body mass index
SCFA: Short-chain fatty acid
UCSC: University of California Santa Cruz
GGTP: Gamma-glutamyltranspeptidase
LDL: Low-density lipoprotein
HDL: High-density cholesterol
PCA: Principal component analysis
UTR: Untranslated region
TSS: Transcriptional start site
ML: Machine learning
