# Supplementary material for: Identified in blood diet-related methylation changes stratify liver biopsies of NAFLD patients according to fibrosis grade
Source: Clin Epigenetics. 2022 Nov 30;14:157. doi: 10.1186/s13148-022-01377-6 (PMC9710135; doi:10.1186/s13148-022-01377-6)
Supplement: Supplementary file 1 — Additional file 1. Supplementary Methods and Results. [file 13148_2022_1377_MOESM1_ESM.docx]

**Supplementary Methods**

**1.** **Identyfication of the most informative CpG sites**

Initial analysis of the genome wide methylation data showed that the methylation in blood of participants changed the most between first and the second timepoint of the study and methylation change between second and third was relieve small. Thus, to simplify data analysis we first for each patient calculated an average methylation level (β-value) from second and third timepoint timepoints of the study and subtracted this average β-value from the β-value of the same CpG site at first timepoint.

We only considered for further analyses CpG sites with more than 10% methylation difference between β-value observed at the first timepoint and the average β-value from second and third timepoint (p-value at less than 9 x 10^-8^ (threshold recently shown as most relevant for this type of studies(12)) and present in at least 3/4 (77%) of participants. That procedure selected a subset of 11,627 CpGs, for which cumulative proportion of variance in PCA analysis reached 83%, and increased to 87% after including data form healthy controls in PCA (**Figure 3B** in main text).

To validate the accuracy of the above procedure to select the subset of the CpG sites with the most informative methylation changes, we randomly selected 100k times the same 11,627 CpGs from entire processed dataset of 666,589 CpGs in our experiment and simulated for each of those subsets a Fowlkes-Mallows Index. FM score is a metric of clustering performance in rage from 0 to 1 and high value indicate a good similarity between real and predicted clusters as opposed to low values, which indicate that clustering cannot distinguish phenotype of interest(13).

In that simulation 76,406 random subsets of 11,627 CpGs displayed higher FM score than the set of CpGs we identified, however sum of 10 principal components explained at the very best 60% of variance in those subsets and only for the subset identified by us the sum of 10 principal components explained more than 90% of variance in the dataset (**Supplementary** **Figure 2**). This indicates that the subset of the CpG sites identified by in our study most accurately described methylation changes in our study.

**2. Assessment of classification accuracy**

To check the accuracy with which our set of 11,627 CpGs can classify the NAFLD liver biopsies correctly to Grade0 and Grade3+ groups, we used automated machine learning TPOT software(14). To reduce the number of data dimensions and remove collinearity in the data we used in calculations data after PCA. Due to imbalance in dataset samples (Grade0 n = 217, Grade3+ n = 124), weights were adjusted. Then the samples in this analysis were split into disjoint training and test datasets at a ratio 7:3 using stratified sampling. The models were trained and optimized using default algorithm settings (mutation_rate=0.9, crossover_rate=0.1), except: generations=10, population_size=50, scoring=”roc_auc”, config_dict=”TPOT light”. Each model was also cross-validated using 5-folds. A detailed description of TPOT is available at <http://epistasislab.github.io/tpot/>.

**3. Food frequency questionnaire (FFQ)**

The frequency and portions size of the meals was assessed with Food frequency questionnaire (FFQ)(1), which consisted of 120-item semi-quantitative questions.

The questionnaire included types of foods itemized into seven broad categories: bread / pasta / rice; vegetables; fruit; meat / fish / egg; beverages; dim sum / snacks; soups; and oil / salt / sauces. Additionally, we included in the questionnaire questions addressing: socio-economic factors, age, nutrient supplement use, food behaviours, developed to measure dietary intake over the six months before the study started.

During the initial training all participants completed the one FFQ questionnaire under the supervision of a licensed nutritionist. The food models and food containers were used during the training in attempt to standardise the portion sizes. Moreover, each of the participants was provided with a catalogue of pictures of individual food portions. The energy intake from cooking oil was calculated as described in (2). Quantifying dietary ranks from the FFQ were converted to dietary frequency according to the formula described in(3).

**4.Mediterranean Diet Quality Index (MDQI)**

Total MDQI is a questionnaire developed to quantify adherence to the Mediterranean diet among children and adolescents. This index is internationally accepted scale for the evaluation of adherence to the Mediterranean diet mostly among children and adolescents, but has also found application among the adults and older adults (4). The survey included 16 yes/no questions and the total index ranges from 0 to 12, and the final score is classified into three levels: ≤3, very poor dietary quality, 4–7, improvements needed to adjust the intake to the patterns of the Mediterranean diet, ≥8, ideal Mediterranean diet.

**5. The physical activity assessment**

We used long version of the International Physical Activity Questionnaire (IPAQ) to assess weekly physical activity of the participants(5). The calculation included multiplication of the metabolic equivalents of a task (MET) by the minutes reported for specific task in a week time. The tasks were: occupation, transportation, housework and recreational activity. Physical activity is also reported in minutes/week of moderate intensity PA using the equation, where minutes reported in each PA domain on the IPAQ by the participant are weighted relative to moderate intensity PA:

Minutes/week = 0.825*walking minutes + 1*moderate minutes + 1.375*garden minutes + 1.5*cycling minutes + 2*vigorous minutes

Total PA was categorized as: low (<600 MET/week), moderate (600-3000 MET/week) and high (≥3000 MET/week). This values in time units corresponds to <150 minutes/week, 150-750 minutes/week and ≥750 minutes/week of moderate intensity PA, respectively(6).

**6. Dietary guidelines and recommended sources of macronutrients and liquids**

The patients were instructed not to alter current daily calories intake but only introduce to the diet products recommended by the Mediterranean Diet Foundation. The recommended sources of carbohydrates included products with a low and medium glycaemic index e.g., row vegetables, fruits. The sources of fat included: vegetable fats, with a predominance of olive oil, and butter. Protein sources were: 2 eggs/week, 5-6 portions of lean meat (with visible fat removed, skinless chicken or turkey), fish with high fat content such as salmon, sardines, trout, whitefish, herring, and mackerel (three times a week). The recommended dairy was lean cottage cheese and participants were asked to eat daylily nuts and seeds. Three portions of vegetables and two portions of fruit were advised and daily fluid intake was calculated to be 35 mL/kg.

**7. Inclusion of high-fiber rolls**

Every three days each participant was supplied with a specifically formulated high-fiber rye high-fiber roll to replace bread normally eaten during breakfast and lunch.

The roles repaired by professional bakery and formulated as follows: 6 - 12 g fiber (Tast, Poland), 40% rye flour type 2000 BIO (Juchowo Farm, Poland), vital fiber (20% plantain, 80%, Poland psyllium) BIO (Tast, Poland) natural leaven from the fermentation of rye flour type 2000 (Living Food, Poland), Himalayan salt (Bioveri, Poland).

**8. 24-hour food diary**

At each of the study timepoints one day before visit participants completed a 24-hour food diary followed a licensed nutritionist instructed. The data from 24-hour food diary we used to estimate consumption of pulses, cereals or grains for breakfast, yoghurts and cheese, baked goods or pastries, dairy products, olive oil, nuts, potatoes, pasta and rice.

**9. Liver stiffness and steatosis measurements**

Liver stiffness (VCTE) and steatosis (CAP) measurements were performed by a single observer (MD trained in this technique), using FibroScan® after at least 6 hours of patients’ fasting at two timepoints of the study (at baseline and at third timepoint). Depending on skin to liver capsule distance the measurements were made using both M and XL probes, in the right lobe of the liver, through intercostal spaces, with the patient lying in a dorsal position and the right arm in maximal abduction. The final results of CAP and VCTE were the median value of 10 measurements expressed in dB/m and kPa, respectively. Only results with an interquartile range ≤40% were considered reliable. The steatosis grades were established using the following cut-off values for low, intermediate and high-grade steatosis (S1, S2, S3): 234, 269 and 301 dB/m. The normal range for stiffness is 2 to 7 kPa.

**10. SCFAs analysis in stool**

**10.1. SCFAs extraction**

A 0.5 g faecal sample was suspended in a tube containing 5 mL of water and mixed for 5 min. The pH of suspension was adjusted to 2–3 with 5 M HCl. The sample than was mixed for 10 min. and centrifuged for 20 min. at 5.000 rpm. The supernatant was filtered (Ø 400 µm) and transferred to a chromatographic vial for gas chromatography analyses(7).

**10.2. Gas Chromatography**

Gas chromatography analysis included: acetic acid (C 2:0), propionic acid (C 3:0), isobutyric acid (C 4:0 i), butyric acid (C 4:0 n), isovaleric acid (C 5:0 i) valeric acid (C 5:0 n). The analysis was performed using Agilent Technologies 1260 A GC system with a flame ionization detector (FID) and fused-silica capillary column with a free fatty acid phase (DB-FFAP, 30 m×0.53 mm×0.5 um). The carrier gas was hydrogen at the flow rate equal to 14.4 mL/min. The injection volume was 1 μL and the run time of a single analysis was 17.5 min at 200°C.

**11. SCFAs analysis in plasma**

**11.1. SCFAs extraction**

40µL of plasma was mixed with 80 µL methanol. Then freshly prepared 20 µL of 3NPH and 20 µL of EDC - pyridine solutions were added to the sample. The mixture was incubated in room temperature for 30 min. Next, solution was diluted to 1mL with 15% aqueous acetonitrile, centrifuged and aliquot was injected into apparatus.

**11.2. Liquid Chromatography**

Liquid chromatography analysis included the same SCFAs as gas chromatography and additionally isocaproic acid (C 6:0 i), caproic acid (C 6:0 n), 2-methylbutyric acid (C5) and 3-methylvaleric acid (C6). The analyis was performed using Waters Acquity Ultra Performance Liquid Chromatograph (Waters, Milford, Massachusetts, USA) coupled with Waters TQ-S triple-quadrupole mass spectrometer (Waters, Manchester, UK) and Waters MassLynx software was used. Waters TargetLynx was used to process data (Waters, Manchester, UK). Waters BEH C18 column (1.7µm, 2.1mm x 50 mm) and Waters BEH C18 guard column (1.7µm, 2.1mm x 5 mm) (Waters, Milford, Massachusetts, USA) were used in analytes separation. Mobile phase A consisted of 1mL of formic acid in 1L water, and mobile phase B consisted of 1mL of formic acid in acetonitrile. The flow rate of mobile phase was set at 0.6 mL/min. The column temperature was 60⁰C, the autosampler was kept at 5⁰C. The injection volume was 10µL. The mass spectrometer operated in multiple-reaction monitoring (MRM) - negative electrospray ionization (ESI) mode. For all analysed compounds mass spectrometer optimized settings were as follows: capillary voltage = 2.25 kV, desolvation temperature = 550 ºC, desolvation gas flow = 550 L/h, cone gas flow = 150 L/h, nebuliser gas pressure = 7.0 bar, source temperature = 150 °C. The first MRM transition of each compound served as a quantitative transition, the second as a confirmation transition. The concentration of SCFAs was calculated in reference to calibration standard mix derived from a series of calibrator samples by spiking standard stock solutions into water. Calibration curves for SCFAs were generated by compared a ratio of the peak area of the analyzed compound to the peak of the internal standard against known analyte concentrations. Mean R2 coefficients of the calibration curves from 6 calibrators was not lower than 0.99. The method showed a good intra- and interassay precision (below 10%).

**12. Histone acetylation analysis**

**12.1. Cells preparation**

Mononuclear cells were extracted from peripheral blood sample using LSM lymphocyte separation medium (MP Biomedicals, Inc, USA) by 30-min centrifugation (2000 rpm, 20^0^C). Following separation PBMCs (a mixture of monocytes and lymphocytes) were washed with phosphate-buffered saline (PBS, Biomed-Lublin, Poland) and resuspended in PBS. The cells were counted using haemocytometer and their viability was assessed by a trypan blue exclusion test (Biomed Lublin Poland). Thereafter, the cells were prepared for further staining(8).

**12.2. Acetylation assessment**

Peripheral blood mononuclear cells (PBMCs) were fixed with 4% formaldehyde (in proportion of one volume of cell suspension to one volume of formaldehyde) at 37^0^C for 10 minutes. Next, cells were washed once with PBS, centrifuged (2000 rpm, 20^0^C), and the supernatant was removed. Then, the cells were permeabilized with ice cold BD Phosflow Perm Buffer II (Becton Dickinson, USA) for 30 minutes and washed twice with PBS and resuspended. Anti-Acetyl-Histone H3-PE antibody was used (as previously described in (9) (Milli-Mark, Merck, Germany) for staining, which was performed following the manufacturer protocol. The protocol included incubation for 30-minutes at room temperature with 10 µL of antibody per 5x10^5^ PBMCs at the concentration recommended by the protocol. Then, the fluorescence intensity (measured as mean fluorescence intensity (MFI) values from histograms), reflecting the level of acetylation of cells were measured in the population of lymphocytes and monocytes by LSR II (Becton Dickinson, USA) flow cytometer as previously described in:(10, 11). The cell fractions were determined by gating on a forward (FSC) and site scatter (SSC) dot plot and verified by CD3-FITC and CD14-FITC staining (Becton Dickinson, USA). A minimum of 1x10^5^ cells were analysed per experiment.

**Supplementary Figures**
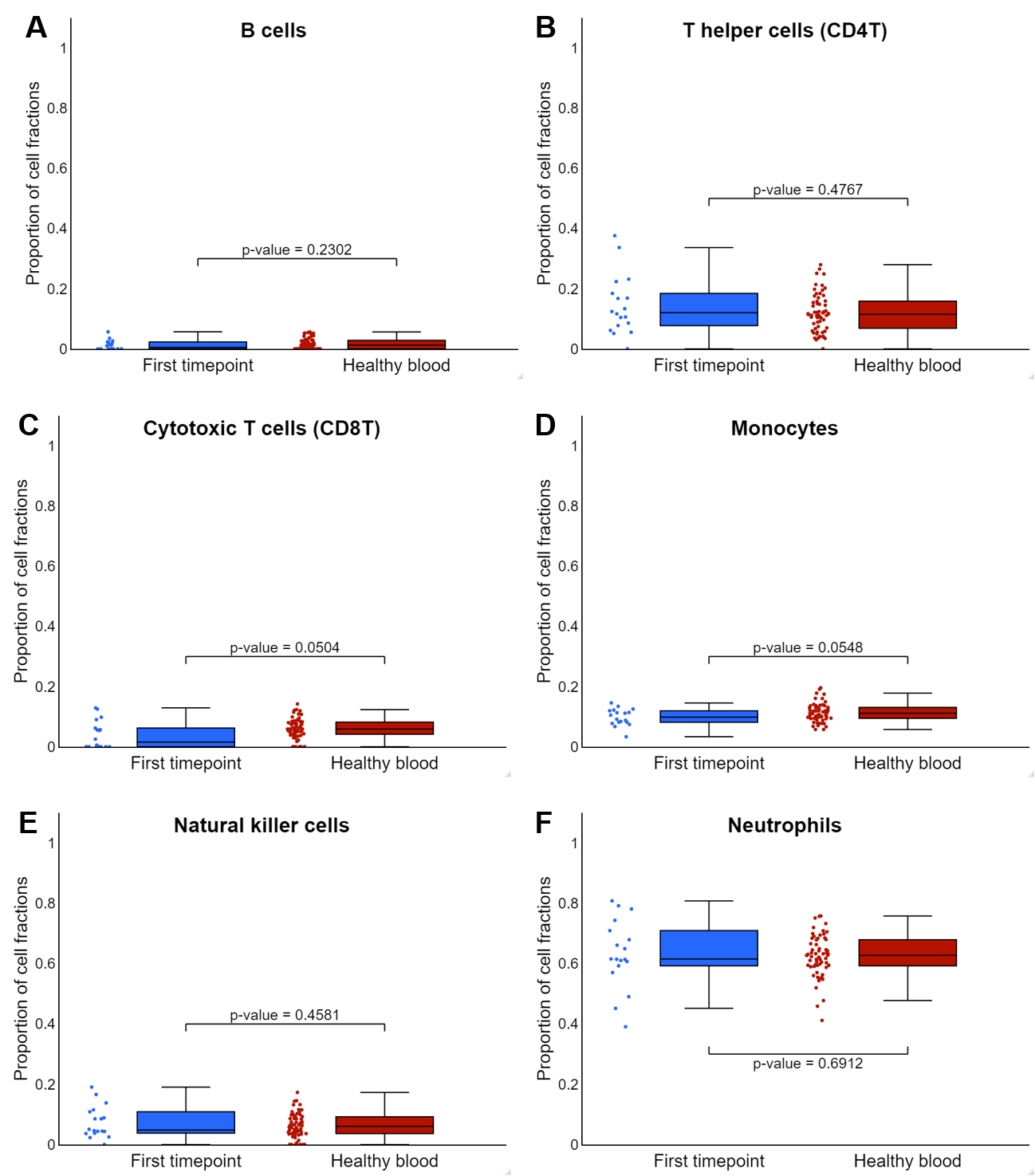


**Supplementary Figure 1.** Comparison of the cell fraction composition in blood of the participants at the first timepoint of the study (blue) and in healthy controls (red). The proportion of cell fractions were calculated using CBS method from EpiDISH package and included: **(A)** B cells, **(B)** T helper cells (CD4T), **(C)** Cytotoxic T cells (CD8T), **(D)** Monocytes, **(E)** Neutrophils, **(F)** Natural killer cells.


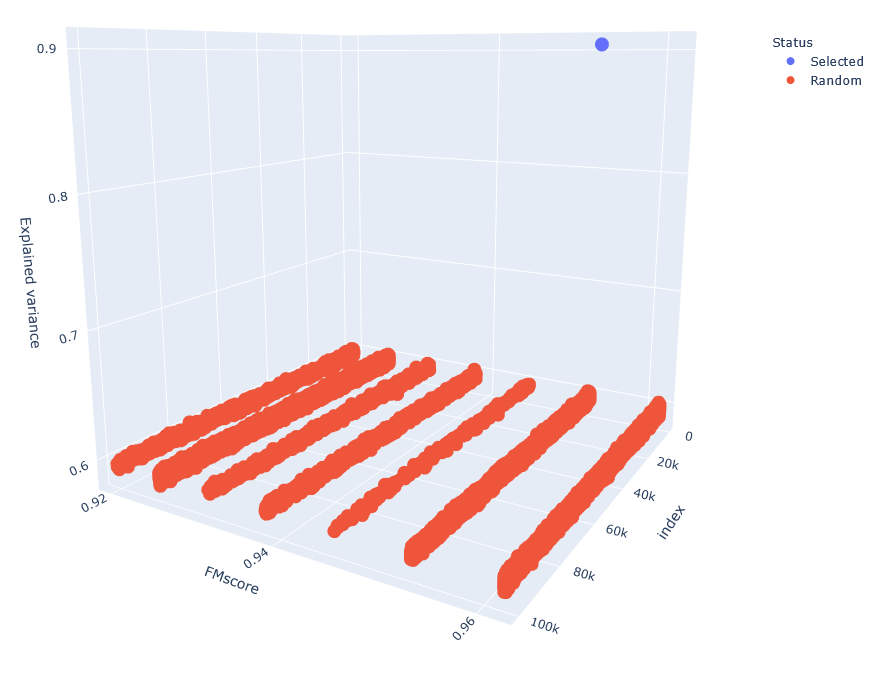


**Supplementary Figure 2. The results of the Fowlkes-Mallows Index simulation.**

Variance determined by PCA is plotted on vertical axis, one of horizontal axis determine the FM score and index, which displays the number of iteration are plotted on horizontal axes. Score for the subset 11,627 CpGs identified in our experiment is marked in purple and each of the randomly selected subset in the simulation are marked in red.


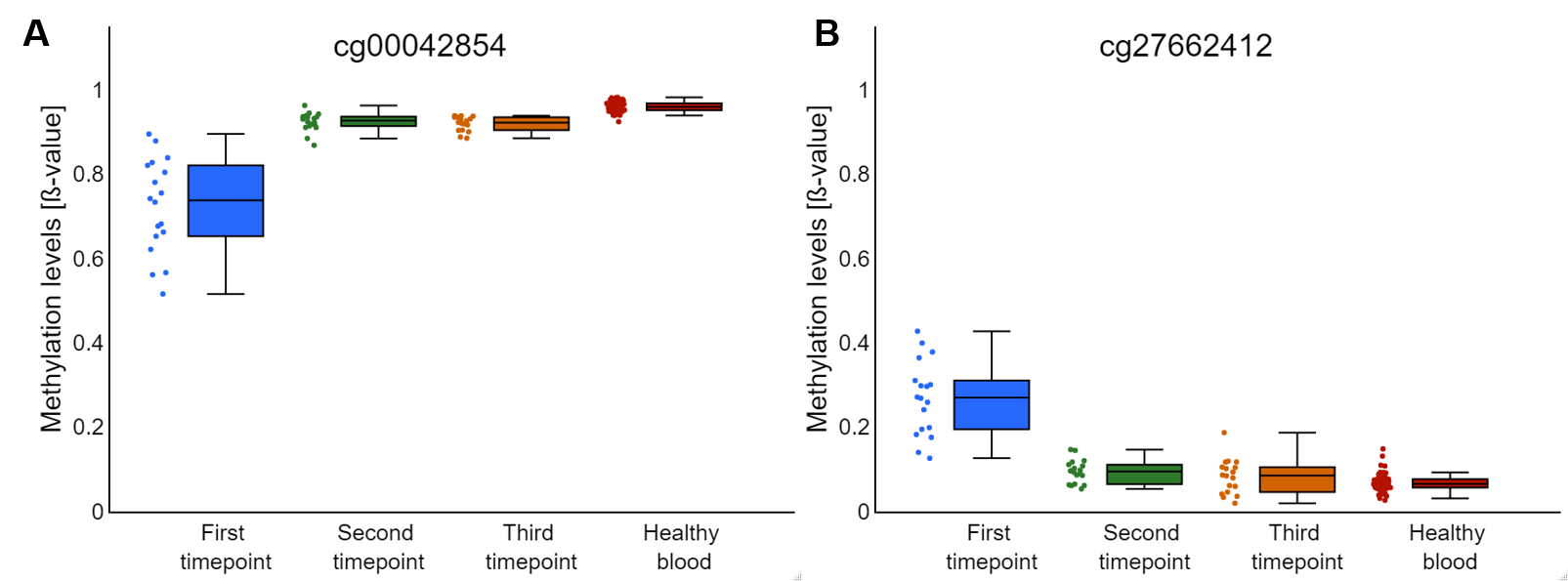


**Supplementary Figure 3.** Examples of the methylation changes observed at specific CpG sites across at each of the study timepoints and in healthy controls. The β-values representing methylation levels are plotted at the vertical axis and the timepoints and healthy blood at the horizontal axis. **(A)** gain of methylation (hypermethylation) in the blood at second and third timepoint, **(B)** loss of methylation (hypomethylation).


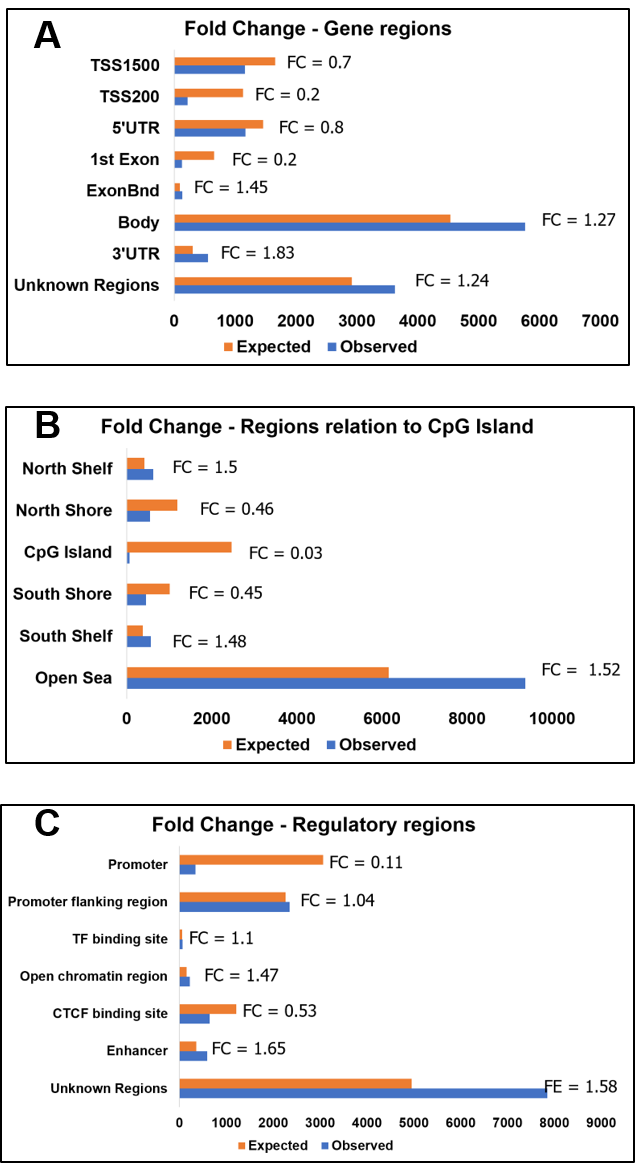


**Supplementary Figure 4.** Analyses of the enrichment of CpG sites with altered methylation levels. Orange bar illustrates expected number of CpGs sites within each region (with 666,589 probes obtained after data processing informative in our study as a background) and blue bar shows observed number of CpGs that were annotated to specific regions including: CpG Island **(A)**, Gene regions **(B)**, Regulatory regions **(C)**.
This analysis indicates that the most affected by the intervention CpG sites are located in regions not directly involved in gene expression regulation including: **(A)** Open Sea, **(B)** ExonBnd, Body, 3’UTR, **(C)** Open chromatin region, Enhancer and Unknown Regions.


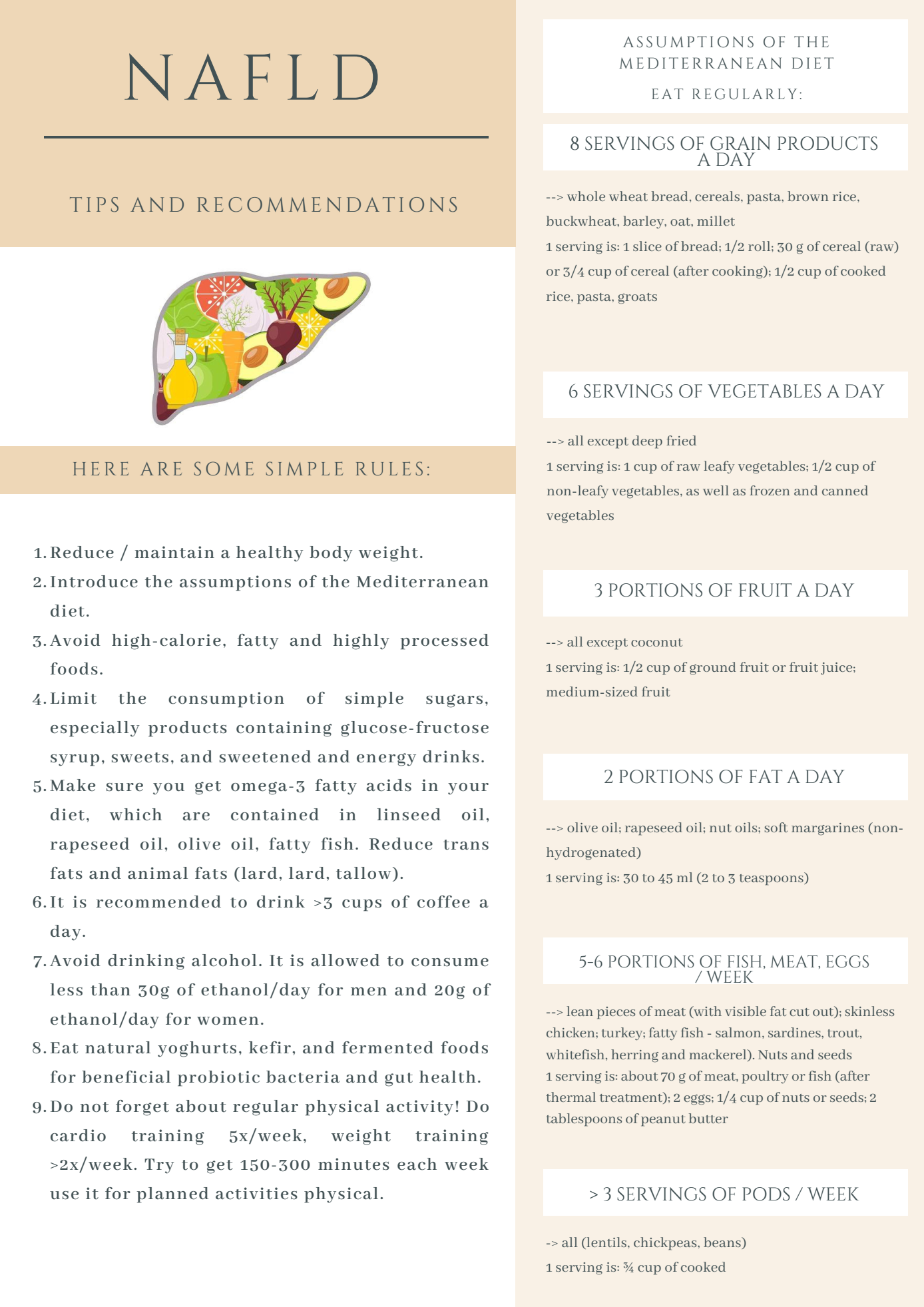


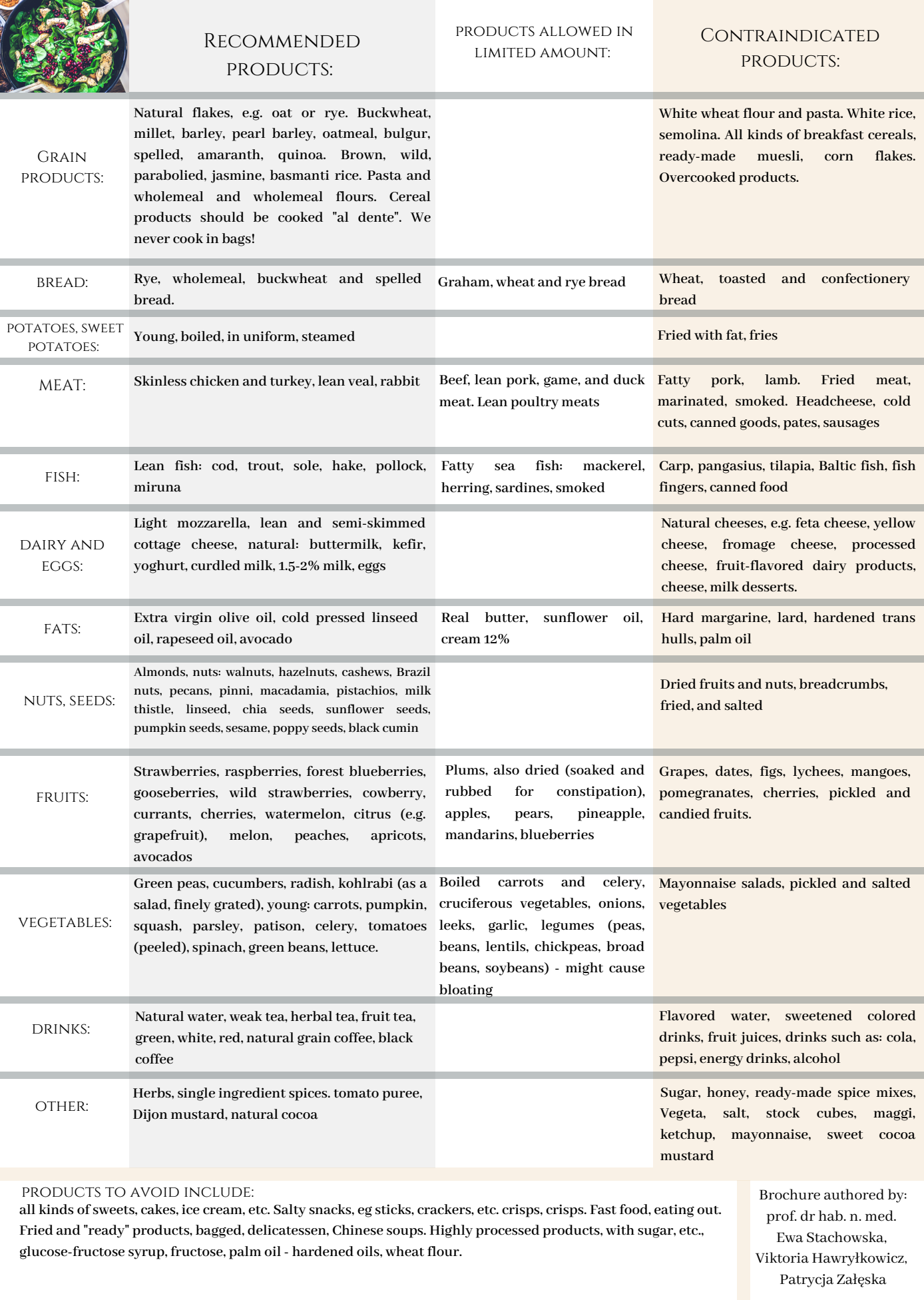


**Supplementary Figure 5.** The pamphlet outlining the dietary instructions that study participants were advised to follow.

**Supplementary Tables**

**Supplementary Table 1. Pearson correlation of global median methylation levels with parameters recorded for participants at inclusion**

| **Parameter** | **Pearson correlation** | **p-value** | **adj. p-value** |
| --- | --- | --- | --- |
| The Mediterranean Diet Quality Index | 0.390958 | 0.108664 | 1 |
| Body weight (kg) | 0.055056 | 0.828227 | 1 |
| BMI (kg/m2) | -0.0934 | 0.712407 | 1 |
| Fat mass (%) | -0.03437 | 0.892291 | 1 |
| Muscle mass (kg) | 0.075795 | 0.765005 | 1 |
| Total Body Water (%) | -0.03103 | 0.90273 | 1 |
| ALT (U/I) | 0.082482 | 0.744897 | 1 |
| AST (U/I) | 0.077122 | 0.761002 | 1 |
| GGTP (U/L) | 0.43139 | 0.073859 | 1 |
| Total Cholesterol (mg/dl) | 0.191366 | 0.446847 | 1 |
| LDL (mg/dl) | 0.227669 | 0.363569 | 1 |
| HDL (mg/dl) | -0.14676 | 0.561161 | 1 |
| Glucose (mg/dL) | -0.04372 | 0.863248 | 1 |
| Insulin (mU/ml) | -0.12401 | 0.623964 | 1 |
| Triglycerides (mg/dl) | -0.0534 | 0.833335 | 1 |
| Liver steatosis - CAP (dB/m) | 0.038961 | 0.878013 | 1 |
| Liver stiffness - VCTE (kPa) | -0.0951 | 0.707377 | 1 |
| Acetic acid (mmol/l) | -0.15972 | 0.526678 | 1 |
| Propionic acid (mmol/l) | -0.29866 | 0.228644 | 1 |
| Isobutyric acid (mmol/l) | -0.55058 | 0.017896 | 0.876884 |
| Butyric acid (mmol/l) | -0.39261 | 0.107045 | 1 |
| Isovaleric acid (mmol/l) | -0.69545 | 0.001353 | 0.066309 |
| Valeric acid (mmol/l) | -0.36025 | 0.141956 | 1 |
| Acetic acid (µM/L) | 0.232517 | 0.353159 | 1 |
| Propionic acid (µM/L) | 0.315295 | 0.2025 | 1 |
| Isobutyric acid (µM/L) | -0.13332 | 0.597934 | 1 |
| Butyric acid (µM/L) | -0.00369 | 0.988399 | 1 |
| 2-Methylbutyric acid (µM/L) | 0.187679 | 0.455814 | 1 |
| Isovaleric acid (µM/L) | -0.36013 | 0.142105 | 1 |
| Valeric acid (µM/L) | 0.24795 | 0.321175 | 1 |
| Isocaproic acid (µM/L) | 0.106878 | 0.67295 | 1 |
| Caproic acid (µM/L) | 0.153673 | 0.542654 | 1 |
| Lymphocyte acetylation (MFI) | 0.351283 | 0.152889 | 1 |
| Monocytes acetylation (MFI) | 0.255434 | 0.306302 | 1 |
| Energy (kcal/day) | 0.369626 | 0.131129 | 1 |
| Fat (g/day) | -0.02271 | 0.928727 | 1 |
| Carbohydrates (g/day) | 0.345922 | 0.15969 | 1 |
| Cholesterol (mg/day) | 0.129435 | 0.608731 | 1 |
| Protein (g/day) | 0.474676 | 0.04654 | 1 |
| Fiber with buns (g/day) | 0.353785 | 0.149783 | 1 |
| Folates (µg/day) | 0.22218 | 0.37556 | 1 |
| Methionine (mg/day) | 0.057817 | 0.819742 | 1 |
| Magnesium (mg/day) | 0.456156 | 0.057083 | 1 |
| Vitamin B12 (mg/day) | 0.504639 | 0.032696 | 1 |
| Vitamin B6 (mg/day) | 0.455881 | 0.057252 | 1 |
| Vitamin C (mg/day) | 0.220574 | 0.379109 | 1 |
| Niacin (vitamin B3) (mg/day) | 0.548259 | 0.018484 | 0.905723 |
| Riboflavin (vitamin B2) (mg/day) | 0.193871 | 0.440806 | 1 |
| Zinc (mg/day) | 0.304753 | 0.21883 | 1 |

**Supplementary Table 2. Pearson correlation of median methylation levels at 11,627 CpGs with the parameter recorded for participants at the first timepoint of the study.**

| **Parameter** | **Pearson correlation** | **p-value** | **adj. p-value** |
| --- | --- | --- | --- |
| The Mediterranean Diet Quality Index | 0.406944 | 0.093734 | 1 |
| Body weight (kg) | 0.052717 | 0.835428 | 1 |
| BMI (kg/m2) | -0.10159 | 0.688352 | 1 |
| Fat mass (%) | -0.0285 | 0.91062 | 1 |
| Muscle mass (kg) | 0.069178 | 0.785041 | 1 |
| Total Body Water (%) | -0.03741 | 0.882835 | 1 |
| ALT (U/I) | -0.03636 | 0.886109 | 1 |
| AST (U/I) | -0.08665 | 0.732456 | 1 |
| GGTP (U/L) | 0.341349 | 0.165651 | 1 |
| Total Cholesterol (mg/dl) | 0.165598 | 0.511378 | 1 |
| LDL (mg/dl) | 0.230681 | 0.357082 | 1 |
| HDL (mg/dl) | -0.13199 | 0.601627 | 1 |
| Glucose (mg/dL) | -0.04225 | 0.867799 | 1 |
| Insulin (mU/ml) | -0.16325 | 0.517472 | 1 |
| Triglycerides (mg/dl) | -0.14686 | 0.560885 | 1 |
| Liver steatosis - CAP (dB/m) | -0.03996 | 0.874913 | 1 |
| Liver stiffness - VCTE (kPa) | -0.13606 | 0.590361 | 1 |
| Acetic acid (mmol/l) | -0.16226 | 0.520039 | 1 |
| Propionic acid (mmol/l) | -0.34039 | 0.166923 | 1 |
| Isobutyric acid (mmol/l) | -0.52551 | 0.025109 | 1 |
| Butyric acid (mmol/l) | -0.43765 | 0.069316 | 1 |
| Isovaleric acid (mmol/l) | -0.62267 | 0.00578 | 0.283242 |
| Valeric acid (mmol/l) | -0.42979 | 0.075055 | 1 |
| Acetic acid (µM/L) | 0.355153 | 0.148104 | 1 |
| Propionic acid (µM/L) | 0.21874 | 0.383186 | 1 |
| Isobutyric acid (µM/L) | -0.28327 | 0.254672 | 1 |
| Butyric acid (µM/L) | -0.03973 | 0.875636 | 1 |
| 2-Methylbutyric acid (µM/L) | 0.197968 | 0.431018 | 1 |
| Isovaleric acid (µM/L) | -0.482 | 0.042807 | 1 |
| Valeric acid (µM/L) | 0.247982 | 0.321111 | 1 |
| Isocaproic acid (µM/L) | 0.01064 | 0.966576 | 1 |
| Caproic acid (µM/L) | 0.153074 | 0.544244 | 1 |
| Lymphocyte acetylation (MFI) | 0.225465 | 0.368357 | 1 |
| Monocytes acetylation (MFI) | -0.00233 | 0.992687 | 1 |
| Energy (kcal/day) | 0.379779 | 0.120064 | 1 |
| Fat (g/day) | 0.161198 | 0.522819 | 1 |
| Carbohydrates (g/day) | 0.145552 | 0.564429 | 1 |
| Cholesterol (mg/day) | -0.05175 | 0.838412 | 1 |
| Protein (g/day) | 0.477344 | 0.045152 | 1 |
| Fiber with buns (g/day) | 0.170189 | 0.49957 | 1 |
| Folates (µg/day) | 0.112874 | 0.655644 | 1 |
| Methionine (mg/day) | 0.150438 | 0.551281 | 1 |
| Magnesium (mg/day) | 0.575492 | 0.012455 | 0.610289 |
| Vitamin B12 (mg/day) | 0.429937 | 0.074945 | 1 |
| Vitamin B6 (mg/day) | 0.300726 | 0.225285 | 1 |
| Vitamin C (mg/day) | 0.126322 | 0.617446 | 1 |
| Niacin (vitamin B3) (mg/day) | 0.414873 | 0.086907 | 1 |
| Riboflavin (vitamin B2) (mg/day) | 0.025013 | 0.921521 | 1 |
| Zinc (mg/day) | 0.169224 | 0.502042 | 1 |

**Supplementary Table 3.** ChIP-Atlas predicts proteins bound to given genomic loci and genes(15). For this analysis we set Antigen class: Histone, Cell class: Blood. As a foreground we used loci of 11,627 CpG sites and as background the loci of 666,589 CpGs that was a result of processed and normalized data. Below table shows results for which Fold Enrichment were greater than 2.

| **ID** | **Antigen** | **Cell** | **Num of peaks** | **Overlaps / input 11627** | **Overlaps / BG** | **Log P-val** | **Log Q-val** | **Fold Enrichment** |
| --- | --- | --- | --- | --- | --- | --- | --- | --- |
| SRX067528 | H3K9me1 | K-562 | 439 | 1/11627 | 3/666589 | -1.17502 | -0.99833 | 19.1104 |
| SRX1802105 | H3K4me3 | Monocytes-CD14+ | 75 | 1/11627 | 6/666589 | -0.94307 | -0.7704 | 9.55519 |
| SRX679458 | H2A.XS139ph | K-562 | 544 | 1/11627 | 8/666589 | -0.84126 | -0.67229 | 7.16639 |
| SRX005148 | H3K4me1 | Hematopoietic Stem Cells | 417 | 1/11627 | 9/666589 | -0.79916 | -0.63092 | 6.37013 |
| SRX6944763 | H3K4me3 | CD4-Positive T-Lymphocytes | 199 | 1/11627 | 9/666589 | -0.79916 | -0.63092 | 6.37013 |
| SRX106083 | H3K27me3 | Lymphoblastoid cells | 43893 | 9/11627 | 85/666589 | -4.43711 | -4.22061 | 6.07035 |
| SRX1802099 | H3K4me3 | Monocytes-CD14+ | 157 | 1/11627 | 10/666589 | -0.76141 | -0.59361 | 5.73311 |
| SRX6944750 | H3K4me3 | CD4-Positive T-Lymphocytes | 76 | 1/11627 | 10/666589 | -0.76141 | -0.59361 | 5.73311 |
| SRX6944771 | H3K4me3 | CD4-Positive T-Lymphocytes | 152 | 1/11627 | 11/666589 | -0.72725 | -0.56106 | 5.21192 |
| SRX6944777 | H3K4me3 | CD4-Positive T-Lymphocytes | 89 | 1/11627 | 11/666589 | -0.72725 | -0.56106 | 5.21192 |
| SRX106086 | H3K27me3 | Lymphoblastoid cells | 26057 | 3/11627 | 36/666589 | -1.536 | -1.35297 | 4.77759 |
| SRX2320913 | H3K4me1 | CD4-Positive T-Lymphocytes | 81 | 1/11627 | 12/666589 | -0.6961 | -0.53211 | 4.77759 |
| SRX6944740 | H3K4me3 | CD4-Positive T-Lymphocytes | 138 | 1/11627 | 12/666589 | -0.6961 | -0.53211 | 4.77759 |
| SRX6944746 | H3K4me3 | CD4-Positive T-Lymphocytes | 344 | 1/11627 | 12/666589 | -0.6961 | -0.53211 | 4.77759 |
| SRX6944754 | H3K4me3 | CD4-Positive T-Lymphocytes | 284 | 1/11627 | 12/666589 | -0.6961 | -0.53211 | 4.77759 |
| SRX6944769 | H3K4me3 | CD4-Positive T-Lymphocytes | 433 | 1/11627 | 12/666589 | -0.6961 | -0.53211 | 4.77759 |
| SRX028593 | H3K9me3 | K-562 | 837 | 1/11627 | 13/666589 | -0.66753 | -0.5044 | 4.41009 |
| SRX1802101 | H3K4me3 | Monocytes-CD14+ | 154 | 1/11627 | 13/666589 | -0.66753 | -0.5044 | 4.41009 |
| SRX6944742 | H3K4me3 | CD4-Positive T-Lymphocytes | 162 | 1/11627 | 13/666589 | -0.66753 | -0.5044 | 4.41009 |
| SRX6944758 | H3K4me3 | CD4-Positive T-Lymphocytes | 213 | 1/11627 | 13/666589 | -0.66753 | -0.5044 | 4.41009 |
| SRX6944756 | H3K4me3 | CD4-Positive T-Lymphocytes | 297 | 1/11627 | 15/666589 | -0.61672 | -0.45418 | 3.82208 |
| SRX6944762 | H3K4me3 | CD4-Positive T-Lymphocytes | 195 | 1/11627 | 15/666589 | -0.61672 | -0.45418 | 3.82208 |
| SRX6944767 | H3K4me3 | CD4-Positive T-Lymphocytes | 247 | 1/11627 | 15/666589 | -0.61672 | -0.45418 | 3.82208 |
| SRX6944775 | H3K4me3 | CD4-Positive T-Lymphocytes | 155 | 1/11627 | 15/666589 | -0.61672 | -0.45418 | 3.82208 |
| SRX106085 | H3K27me3 | Lymphoblastoid cell line | 22554 | 2/11627 | 31/666589 | -0.96049 | -0.78754 | 3.69878 |
| SRX3478256 | H2A.XS139ph | K-562 | 777 | 1/11627 | 16/666589 | -0.59397 | -0.43171 | 3.5832 |
| SRX2402112 | H3K9me3 | HL-60 | 596 | 2/11627 | 34/666589 | -0.89804 | -0.72789 | 3.37242 |
| SRX3164076 | H3K4me1 | K-562 | 280 | 2/11627 | 34/666589 | -0.89804 | -0.72789 | 3.37242 |
| SRX1802103 | H3K4me3 | Monocytes-CD14+ | 90 | 1/11627 | 17/666589 | -0.57271 | -0.41089 | 3.37242 |
| SRX6944744 | H3K4me3 | CD4-Positive T-Lymphocytes | 297 | 1/11627 | 18/666589 | -0.55279 | -0.39571 | 3.18506 |
| SRX6944779 | H3K4me3 | CD4-Positive T-Lymphocytes | 422 | 1/11627 | 18/666589 | -0.55279 | -0.39571 | 3.18506 |
| SRX1802109 | H3K4me3 | Monocytes-CD14+ | 146 | 1/11627 | 19/666589 | -0.53405 | -0.37798 | 3.01743 |
| SRX6944752 | H3K4me3 | CD4-Positive T-Lymphocytes | 361 | 1/11627 | 19/666589 | -0.53405 | -0.37798 | 3.01743 |
| SRX3164075 | H3K4me1 | K-562 | 239 | 1/11627 | 20/666589 | -0.5164 | -0.3609 | 2.86656 |
| SRX6944760 | H3K4me3 | CD4-Positive T-Lymphocytes | 406 | 1/11627 | 20/666589 | -0.5164 | -0.3609 | 2.86656 |
| SRX1802107 | H3K4me3 | Monocytes-CD14+ | 294 | 1/11627 | 22/666589 | -0.48392 | -0.32885 | 2.60596 |
| SRX1802115 | H3K4me3 | Monocytes-CD14+ | 99 | 1/11627 | 22/666589 | -0.48392 | -0.32885 | 2.60596 |
| SRX3796991 | H3K4me3 | Monocytes-CD14+ | 110 | 1/11627 | 22/666589 | -0.48392 | -0.32885 | 2.60596 |
| SRX106084 | H3K27me3 | Lymphoblastoid cell line | 16517 | 1/11627 | 23/666589 | -0.46894 | -0.31444 | 2.49266 |
| SRX3796997 | H3K4me3 | Monocytes-CD14+ | 956 | 1/11627 | 23/666589 | -0.46894 | -0.31444 | 2.49266 |
| SRX3796993 | H3K4me3 | Monocytes-CD14+ | 116 | 1/11627 | 24/666589 | -0.4547 | -0.30048 | 2.3888 |
| SRX190085 | H3K36me3 | K-562 | 209 | 1/11627 | 27/666589 | -0.41589 | -0.26281 | 2.12338 |
| ERX3216998 | H3K4me1 | Lymphoblastoid cell line | 661 | 2/11627 | 55/666589 | -0.59216 | -0.43005 | 2.08477 |
| ERX3216873 | H3K4me1 | Lymphoblastoid cell line | 9699 | 18/11627 | 510/666589 | -2.20031 | -2.00869 | 2.02345 |
| SRX3586324 | H3K4me1 | Macrophages | 405 | 2/11627 | 57/666589 | -0.57108 | -0.4094 | 2.01162 |

**Supplementary Table 4.** Motif enrichment was calculated via HOMER (v4.11)(16). This tool searches for de novo motif matches that are enriched in a set of foreground sequences relative to a given set of background sequences. The set of 11,627 CpG sites that identified in our analyses was a foreground and 666,589 probes informative across all the samples in the study, was a background in this analysis. We used parameters such as masking and hypergeometric enrichment calculations and the size of 200 bp in the analysis. Below only results with equal and lower than 1e-12 p-value are presented (as HOMER recommended).

| **Rank** | **Motif** | **Score** | **p-value** | **% of Targets** | **% of Background** | **Best Match/Details** |
| --- | --- | --- | --- | --- | --- | --- |
| 1 | 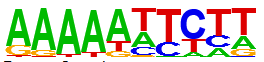 | 0.69 | 1e-23 | 18.65% | 14.68% | ZNF384/MA1125.1/Jaspar |
| 2 | 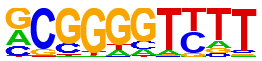 | 0.7 | 1e-20 | 9.16% | 6.51% | LRF(Zf)/Erythroblasts-ZBTB7A-ChIP-Seq(GSE74977)/Homer |
| 3 | 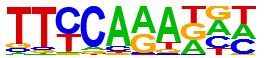 | 0.75 | 1e-13 | 6.25% | 4.49% | ZBTB26/MA1579.1/Jaspar |
| 4 | 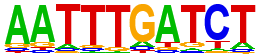 | 0.76 | 1e-13 | 13.86% | 11.27% | PHOX2B/MA0681.2/Jaspar |
| 5 | 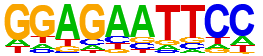 | 0.73 | 1e-12 | 22.26% | 19.21% | RELB/MA1117.1/Jaspar |
| 6 | 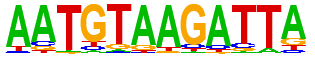 | 0.64 | 1e-12 | 2.27% | 1.32% | GATA1/MA0035.4/Jaspar |

**Supplementary Table 5. GSEA – FUMA**

FUMA is a tool which allows to use functional, biological information to prioritize genes sets and we used this platform to perform gene set enrichment analyses of the genes annotated to 11,627 CpGs identified in our study. First we assessed the tissue specific enrichment of those genes and **Supplementary Table 5A** shows the results of this analysis. Then, we integrated nine gene set databases including: Hallmark gene sets, Curated gene sets, Chemical and Genetic perturbation gene sets (MsigDB c2), All Canonical Pathways (MsigDB c2), KEGG (MsigDB c2), Reactome (MsigDB c2), GO biological processes (MsigDB c5), GO cellular components (MsigDB c5), GO molecular functions (MsigDB c5) for enrichment of the gene set from our study in specific ontology terms. The results of this analysis are listed in **Supplementary Table 5B** (the tables show 20 top hits for the searches that returned more than 20 terms). The publication searched in Google Scholar were performed on 15-22.11.2021.

**Supplementary Table 5A – Tissue specificity**

| **No.** | **Differentially expressed genes direction** | **Tissue type in the analysis** | **Search term from the analysis in Google Scholar** | **PMID of articles, which were found in Google S**cholar **search** |
| --- | --- | --- | --- | --- |
| 1 | down-regulated | Liver | Liver + NAFLD | NAFLD is a liver disease, so we did not search for articles |
| 2 | down-regulated | Kidney_Cortex | Kidney Cortex + NAFLD | PMID: **32059982,** PMID: **31738141,** PMID: **30912854** |
| 3 | up-regulated | Artery_Tibial | Artery Tibial + NAFLD | Found articles are mainly focused on arterial stiffness, atherosclerosis: PMID: **22986520,** PMID: **28696562,** PMID: **33990287** |
| 4 | up-regulated | Nerve_Tibial | Nerve Tibial + NAFLD | No results, however term “Nerve Tibial” suggest the association with nervous system and for search we used “Nerve + NAFLD” and found: PMID: **33149804,** PMID: **33787507,** PMID: **31278754** |

**Supplementary Table 5B – Ontology Terms**

**Database I - Hallmark gene sets**

| **No.** | **GeneSet** | **Google Scholar search term*** | **Results from Google Scholar searching - PMID** | **Total number of genes related to the “GeneSet” term** | **Number of genes from our input gene set, that overlap with genes related to the “GeneSet” term** | **Enrichment P-value** | **Adjusted**  **P-value** |
| --- | --- | --- | --- | --- | --- | --- | --- |
| 1 | HALLMARK UV RESPONSE DN | UV RESPONSE DN + NAFLD | PMID: 25544877 | 142 | 80 | 7.62E-16 | 3.81E-14 |
| 2 | HALLMARK MITOTIC SPINDLE | MITOTIC SPINDLE + NAFLD | No results | 189 | 92 | 7.69E-13 | 1.92E-11 |
| 3 | HALLMARK ANDROGEN RESPONSE | ANDROGEN RESPONSE + NAFLD | PMID: **34572151,**  PMID: **32294235,** PMID: **23721173,** PMID: **34360667** | 97 | 43 | 1.89E-05 | 0.000315 |
| 4 | HALLMARK PROTEIN SECRETION | PROTEIN SECRETION + NAFLD | PMID: **31098621** | 88 | 37 | 0.000263 | 0.003086 |
| 5 | HALLMARK IL2 STAT5 SIGNALING | IL2 STAT5 SIGNALING + NAFLD | PMID: 30787925 | 195 | 70 | 0.000309 | 0.003086 |
| 6 | HALLMARK TGF BETA SIGNALING | TGF BETA SIGNALING + NAFLD | PMID: **30572631,** PMID: **33768585,** PMID: **30582764** | 53 | 24 | 0.00087 | 0.007253 |
| 7 | HALLMARK KRAS SIGNALING UP | KRAS SIGNALING UP + NAFLD | PMID: **32947972,** PMID: **32001554** | 191 | 65 | 0.002351 | 0.016791 |
| 8 | HALLMARK HEME METABOLISM | HEME METABOLISM + NAFLD | PMID: **33276146**,  PMID: **33327438** | 186 | 62 | 0.004951 | 0.030945 |

*** “HALMARK” ” – the name of database, were removed from the Google Scholar search term**

**Database II – Curated gene sets**

| **No.** | **GeneSet** | **Google Scholar search term*** | **Results from Google Scholar searching - PMID** | **Total number of genes related to the “GeneSet” term** | **Number of genes from our input gene set, that overlap with genes related to the “GeneSet” term** | **Enrichment P-value** | **Adjusted**  **P-value** |
| --- | --- | --- | --- | --- | --- | --- | --- |
| 1 | DACOSTA UV RESPONSE VIA ERCC3 DN | UV RESPONSE VIA ERCC3 DN + NAFLD | No results | 835 | 490 | 3.28E-101 | 1.80E-97 |
| 2 | DACOSTA UV RESPONSE VIA ERCC3 COMMON DN | UV RESPONSE VIA ERCC3 COMMON DN + NAFLD | No results | 453 | 286 | 1.50E-68 | 4.13E-65 |
| 3 | GRAESSMANN APOPTOSIS BY DOXORUBICIN DN | APOPTOSIS BY DOXORUBICIN DN + NAFLD | PMID: **29290620** | 1616 | 626 | 6.20E-39 | 1.14E-35 |
| 4 | CUI TCF21 TARGETS 2 DN | TCF21 TARGETS 2 + NAFLD | No results | 777 | 353 | 1.09E-37 | 1.50E-34 |
| 5 | GRYDER PAX3FOXO1 ENHANCERS IN TADS | PAX3FOXO1 ENHANCERS IN TADS + NAFLD | No results | 968 | 405 | 3.19E-33 | 3.51E-30 |
| 6 | HAMAI APOPTOSIS VIA TRAIL UP | APOPTOSIS VIA TRAIL UP + NAFLD | PMID: 29574534,  PMID: 21629127 | 621 | 275 | 2.72E-27 | 2.49E-24 |
| 7 | ZHENG BOUND BY FOXP3 | BOUND BY FOXP3 + NAFLD | PMID: **31605031** | 460 | 218 | 1.43E-26 | 1.12E-23 |
| 8 | PILON KLF1 TARGETS DN | KLF1 TARGETS DN + NAFLD | No results | 1878 | 645 | 5.21E-23 | 3.58E-20 |
| 9 | GABRIELY MIR21 TARGETS | MIR21 TARGETS + NAFLD | PMID: 26282675, PMID: **27436271** | 272 | 142 | 1.13E-22 | 6.89E-20 |
| 10 | DUTERTRE ESTRADIOL RESPONSE 24HR DN | ESTRADIOL RESPONSE 24HR DN + NAFLD | PMID: **31589598** | 483 | 214 | 1.68E-21 | 9.25E-19 |
| 11 | RODRIGUES THYROID CARCINOMA POORLY DIFFERENTIATED DN | THYROID CARCINOMA POORLY DIFFERENTIATED DN + NAFLD | No results of thyroid carcinoma and NAFLD, but there are articles associated thyroid function with NAFLD:  PMID: **25330278** | 734 | 293 | 1.66E-20 | 8.29E-18 |
| 12 | DAZARD RESPONSE TO UV NHEK DN | RESPONSE TO UV NHEK DN + NAFLD | No results | 293 | 145 | 2.92E-20 | 1.34E-17 |
| 13 | GOBERT OLIGODENDROCYTE DIFFERENTIATION DN | OLIGODENDROCYTE DIFFERENTIATION DN + NAFLD | No results | 1005 | 372 | 4.14E-19 | 1.75E-16 |
| 14 | GOZGIT ESR1 TARGETS DN | ESR1 TARGETS DN + NAFLD | PMID: **34572151** | 686 | 270 | 5.12E-18 | 2.01E-15 |
| 15 | DAZARD UV RESPONSE CLUSTER G6 | UV RESPONSE CLUSTER G6 + NAFLD | No results | 141 | 83 | 5.70E-18 | 2.09E-15 |
| 16 | CREIGHTON ENDOCRINE THERAPY RESISTANCE 5 | ENDOCRINE THERAPY RESISTANCE 5 + NAFLD | PMID: **32147363** | 454 | 195 | 6.56E-18 | 2.25E-15 |
| 17 | GRYDER PAX3FOXO1 ENHANCERS KO DOWN | PAX3FOXO1 ENHANCERS KO DOWN + NAFLD | No results | 425 | 185 | 8.90E-18 | 2.88E-15 |
| 18 | ZHENG FOXP3 TARGETS IN THYMUS UP | FOXP3 TARGETS IN THYMUS UP + NAFLD | PMID: **31991386,** PMID: 29062902 | 188 | 101 | 1.17E-17 | 3.57E-15 |
| 19 | SCHLOSSER SERUM RESPONSE DN | SERUM RESPONSE DN + NAFLD | PMID: **29456698** | 660 | 260 | 1.90E-17 | 5.49E-15 |
| 20 | IKEDA MIR30 TARGETS UP | MIR30 TARGETS UP + NAFLD | PMID: **32419060** | 110 | 69 | 3.14E-17 | 8.64E-15 |

***** **Author surname of the source publication was removed from the Google Scholar search term**

**Database III – Chemical and Genetic perturbation**

| **No.** | **GeneSet** | **Google Scholar search term*** | **Results from Google Scholar searching - PMID** | **Total number of genes related to the “GeneSet” term** | **Number of genes from our input gene set, that overlap with genes related to the “GeneSet” term** | **Enrichment P-value** | **Adjusted**  **P-value** |
| --- | --- | --- | --- | --- | --- | --- | --- |
| 1 | DACOSTA UV RESPONSE VIA ERCC3 DN | UV RESPONSE VIA ERCC3 DN + NAFLD | No results | 835 | 490 | 3.28E-101 | 1.08E-97 |
| 2 | DACOSTA UV RESPONSE VIA ERCC3 COMMON DN | UV RESPONSE VIA ERCC3 COMMON DN + NAFLD | No results | 453 | 286 | 1.50E-68 | 2.48E-65 |
| 3 | GRAESSMANN APOPTOSIS BY DOXORUBICIN DN | APOPTOSIS BY DOXORUBICIN DN + NAFLD | PMID: **29290620** | 1616 | 626 | 6.20E-39 | 6.82E-36 |
| 4 | CUI TCF21 TARGETS 2 DN | TCF21 TARGETS 2 + NAFLD | No results | 777 | 353 | 1.09E-37 | 8.99E-35 |
| 5 | GRYDER PAX3FOXO1 ENHANCERS IN TADS | PAX3FOXO1 ENHANCERS IN TADS + NAFLD | No results | 968 | 405 | 3.19E-33 | 2.11E-30 |
| 6 | HAMAI APOPTOSIS VIA TRAIL UP | APOPTOSIS VIA TRAIL UP + NAFLD | PMID: 29574534,  PMID: 21629127 | 621 | 275 | 2.72E-27 | 1.50E-24 |
| 7 | ZHENG BOUND BY FOXP3 | BOUND BY FOXP3 + NAFLD | PMID: **31605031** | 460 | 218 | 1.43E-26 | 6.74E-24 |
| 8 | PILON KLF1 TARGETS DN | KLF1 TARGETS DN + NAFLD | No results | 1878 | 645 | 5.21E-23 | 2.15E-20 |
| 9 | GABRIELY MIR21 TARGETS | MIR21 TARGETS + NAFLD | PMID: 26282675, PMID: **27436271** | 272 | 142 | 1.13E-22 | 4.14E-20 |
| 10 | DUTERTRE ESTRADIOL RESPONSE 24HR DN | ESTRADIOL RESPONSE 24HR DN + NAFLD | PMID: **29391015** | 483 | 214 | 1.68E-21 | 5.55E-19 |
| 11 | RODRIGUES THYROID CARCINOMA POORLY DIFFERENTIATED DN | THYROID CARCINOMA POORLY DIFFERENTIATED DN + NAFLD | no results of thyroid carcinoma and NAFLD, but there are articles associated thyroid function with NAFLD: PMID: **25330278** | 734 | 293 | 1.66E-20 | 4.98E-18 |
| 12 | DAZARD RESPONSE TO UV NHEK DN | RESPONSE TO UV NHEK DN + NAFLD | No results | 293 | 145 | 2.92E-20 | 8.03E-18 |
| 13 | GOBERT OLIGODENDROCYTE DIFFERENTIATION DN | OLIGODENDROCYTE DIFFERENTIATION DN + NAFLD | No results | 1005 | 372 | 4.14E-19 | 1.05E-16 |
| 14 | GOZGIT ESR1 TARGETS DN | ESR1 TARGETS DN + NAFLD | PMID: **34572151** | 686 | 270 | 5.12E-18 | 1.21E-15 |
| 15 | DAZARD UV RESPONSE CLUSTER G6 | UV RESPONSE CLUSTER G6 + NAFLD | No results | 141 | 83 | 5.70E-18 | 1.25E-15 |
| 16 | CREIGHTON ENDOCRINE THERAPY RESISTANCE 5 | ENDOCRINE THERAPY RESISTANCE 5 + NAFLD | PMID: **32147363** | 454 | 195 | 6.56E-18 | 1.35E-15 |
| 17 | GRYDER PAX3FOXO1 ENHANCERS KO DOWN | PAX3FOXO1 ENHANCERS KO DOWN + NAFLD | No results | 425 | 185 | 8.90E-18 | 1.73E-15 |
| 18 | ZHENG FOXP3 TARGETS IN THYMUS UP | FOXP3 TARGETS IN THYMUS UP + NAFLD | PMID: **31991386,** PMID: 29062902 | 188 | 101 | 1.17E-17 | 2.14E-15 |
| 19 | SCHLOSSER SERUM RESPONSE DN | SERUM RESPONSE DN + NAFLD | PMID: **29456698** | 660 | 260 | 1.90E-17 | 3.30E-15 |
| 20 | IKEDA MIR30 TARGETS UP | MIR30 TARGETS UP + NAFLD | PMID: **32419060** | 110 | 69 | 3.14E-17 | 5.18E-15 |

***** **Author surname of the source publication was removed from the Google Scholar search term**

**Database IV – Canonical Pathways**

| **No.** | **GeneSet** | **Google Scholar search term*** | **Results from Google Scholar searching - PMID** | **Total number of genes related to the “GeneSet” term** | **Number of genes from our input gene set, that overlap with genes related to the “GeneSet” term** | **Enrichment P-value** | **Adjusted**  **P-value** |
| --- | --- | --- | --- | --- | --- | --- | --- |
| 1 | KEGG ADHERENS JUNCTION | ADHERENS JUNCTION + NAFLD | PMID: **33193105,** PMID: **26841783,** PMID: 34280073 | 72 | 38 | 2.89E-07 | 0.000521 |
| 2 | REACTOME SIGNALING BY RHO GTPASES | SIGNALING BY RHO GTPASES + NAFLD | PMID: **27634010,** PMID: **29574534** | 419 | 148 | 6.33E-07 | 0.000521 |
| 3 | PID NFAT 3PATHWAY | NFAT 3PATHWAY + NAFLD | PMID: **32717288** | 53 | 30 | 7.10E-07 | 0.000521 |
| 4 | REACTOME RHO GTPASE CYCLE | RHO GTPASE CYCLE + NAFLD | PMID: **31467298** | 127 | 56 | 1.38E-06 | 0.000757 |
| 5 | REACTOME MITOTIC SPINDLE CHECKPOINT | MITOTIC SPINDLE CHECKPOINT + NAFLD | No results | 108 | 49 | 2.28E-06 | 0.001001 |
| 6 | REACTOME SIGNALING BY RECEPTOR TYROSINE KINASES | SIGNALING BY RECEPTOR TYROSINE KINASES + NAFLD | PMID: **29038049,**  PMID: **32372975** | 453 | 155 | 2.94E-06 | 0.001077 |
| 7 | REACTOME NEURONAL SYSTEM | NEURONAL SYSTEM + NAFLD | PMID: **24019886** | 389 | 135 | 5.53E-06 | 0.001738 |
| 8 | REACTOME METABOLISM OF LIPIDS | METABOLISM OF LIPIDS + NAFLD | PMID: **33010471,**  PMID: **26198708** | 702 | 224 | 7.21E-06 | 0.001982 |
| 9 | REACTOME DISEASES OF SIGNAL TRANSDUCTION | DISEASES OF SIGNAL TRANSDUCTION + NAFLD | PMID: 19452573,  PMID: 25126158 | 371 | 128 | 1.32E-05 | 0.003006 |
| 10 | REACTOME EFFECTS OF PIP2 HYDROLYSIS | EFFECTS OF PIP2 HYDROLYSIS + NAFLD | PMID: **27132506** | 26 | 17 | 1.37E-05 | 0.003006 |
| 11 | PID FAK PATHWAY | FAK PATHWAY + NAFLD | PMID: 29221151 | 57 | 29 | 1.83E-05 | 0.003665 |
| 12 | REACTOME MITOTIC PROMETAPHASE | MITOTIC PROMETAPHASE + NAFLD | PMID: **32643289** | 188 | 72 | 2.39E-05 | 0.004379 |
| 13 | BIOCARTA CCR3 PATHWAY | CCR3 PATHWAY + NAFLD | PMID: **32039405** | 18 | 13 | 2.98E-05 | 0.00504 |
| 14 | REACTOME RESOLUTION OF SISTER CHROMATID COHESION | RESOLUTION OF SISTER CHROMATID COHESION + NAFLD | PMID: **33526312,** PMID: **29974848** | 120 | 50 | 3.24E-05 | 0.005069 |
| 15 | REACTOME SIGNALING BY TGF BETA FAMILY MEMBERS | SIGNALING BY TGF BETA FAMILY MEMBERS + NAFLD | PMID: **30582764,**  PMID: **32054379** | 99 | 43 | 3.46E-05 | 0.005069 |
| 16 | REACTOME SUMOYLATION | SUMOYLATION + NAFLD | PMID: 32710938, PMID: **28094767** | 179 | 68 | 5.33E-05 | 0.007016 |
| 17 | REACTOME SIGNALING BY TGF BETA RECEPTOR COMPLEX | SIGNALING BY TGF BETA RECEPTOR COMPLEX + NAFLD | PMID: **32054379,** PMID: **32191345** | 71 | 33 | 5.42E-05 | 0.007016 |
| 18 | REACTOME NETRIN 1 SIGNALING | NETRIN 1 SIGNALING + NAFLD | PMID: 29985430 | 49 | 25 | 6.44E-05 | 0.007865 |
| 19 | REACTOME PROTEIN PROTEIN INTERACTIONS AT SYNAPSES | PROTEIN PROTEIN INTERACTIONS AT SYNAPSES + NAFLD | PMID: **29564062** | 78 | 35 | 8.06E-05 | 0.009326 |
| 20 | REACTOME CELL CYCLE | CELL CYCLE + NAFLD | PMID: **32578019** | 615 | 193 | 8.74E-05 | 0.009609 |

*** “KEGG”, “REACTOME”,”PID”, “**BIOCARTA” **– the names of databases, were removed from the Google Scholar search term**

**Database V – KEGG**

| **No.** | **GeneSet** | **Google Scholar search term*** | **Results from Google Scholar searching - PMID** | **Total number of genes related to the “GeneSet” term** | **Number of genes from our input gene set, that overlap with genes related to the “GeneSet” term** | **Enrichment P-value** | **Adjusted**  **P-value** |
| --- | --- | --- | --- | --- | --- | --- | --- |
| 1 | KEGG ADHERENS JUNCTION | ADHERENS JUNCTION + NAFLD | PMID: **33193105,** PMID: **26841783,** PMID: 34280073 | 72 | 38 | 2.89E-07 | 5.37E-05 |
| 2 | KEGG REGULATION OF ACTIN CYTOSKELETON | REGULATION OF ACTIN CYTOSKELETON + NAFLD | No results | 201 | 73 | 0.000154 | 0.009359 |
| 3 | KEGG LONG TERM POTENTIATION | LONG TERM POTENTIATION + NAFLD | PMID: **28211103** | 66 | 30 | 0.000192 | 0.009359 |
| 4 | KEGG PHOSPHATIDYLINOSITOL SIGNALING SYSTEM | PHOSPHATIDYLINOSITOL SIGNALING SYSTEM + NAFLD | PMID: 33390956 | 75 | 33 | 0.000201 | 0.009359 |
| 5 | KEGG FOCAL ADHESION | FOCAL ADHESION + NAFLD | PMID: **26845596** | 193 | 69 | 0.000388 | 0.014415 |
| 6 | KEGG AXON GUIDANCE | AXON GUIDANCE + NAFLD | PMID: **34435630** | 124 | 47 | 0.000749 | 0.023225 |
| 7 | KEGG ARRHYTHMOGENIC RIGHT VENTRICULAR CARDIOMYOPATHY ARVC | ARRHYTHMOGENIC RIGHT VENTRICULAR CARDIOMYOPATHY ARVC + NAFLD | No results | 71 | 30 | 0.000877 | 0.023311 |
| 8 | KEGG ABC TRANSPORTERS | ABC TRANSPORTERS + NAFLD | PMID: 21878559 | 42 | 20 | 0.001065 | 0.024039 |
| 9 | KEGG WNT SIGNALING PATHWAY | WNT SIGNALING PATHWAY + NAFLD | PMID: 27556491 | 146 | 53 | 0.001163 | 0.024039 |
| 10 | KEGG NUCLEOTIDE EXCISION REPAIR | NUCLEOTIDE EXCISION REPAIR + NAFLD | PMID: **22100520** | 40 | 19 | 0.001464 | 0.027238 |
| 11 | KEGG PATHWAYS IN CANCER | PATHWAYS IN CANCER + NAFLD | PMID: **29956206** | 315 | 101 | 0.001825 | 0.028881 |
| 12 | KEGG DORSO VENTRAL AXIS FORMATION | DORSO VENTRAL AXIS FORMATION + NAFLD | No results | 24 | 13 | 0.001863 | 0.028881 |
| 13 | KEGG LONG TERM DEPRESSION | LONG TERM DEPRESSION + NAFLD | PMID: **32708059** | 68 | 28 | 0.002049 | 0.029313 |
| 14 | KEGG INOSITOL PHOSPHATE METABOLISM | INOSITOL PHOSPHATE METABOLISM + NAFLD | PMID: 33153126 | 53 | 23 | 0.00222 | 0.029493 |
| 15 | KEGG OOCYTE MEIOSIS | OOCYTE MEIOSIS + NAFLD | No results | 107 | 40 | 0.002394 | 0.02969 |
| 16 | KEGG LYSINE DEGRADATION | LYSINE DEGRADATION + NAFLD | PMID: 28745372, PMID: 32586350 | 42 | 19 | 0.002961 | 0.032394 |
| 17 | KEGG VALINE LEUCINE AND ISOLEUCINE DEGRADATION | VALINE LEUCINE AND ISOLEUCINE DEGRADATION + NAFLD | PMID: 25534430 | 42 | 19 | 0.002961 | 0.032394 |
| 18 | KEGG CALCIUM SIGNALING PATHWAY | CALCIUM SIGNALING PATHWAY + NAFLD | PMID: 30850262 | 166 | 57 | 0.003363 | 0.034533 |
| 19 | KEGG TIGHT JUNCTION | TIGHT JUNCTION + NAFLD | PMID: 27366215, PMID: 24895809, PMID: 19291785 | 129 | 46 | 0.003528 | 0.034533 |
| 20 | KEGG FC GAMMA R MEDIATED PHAGOCYTOSIS | FC GAMMA R MEDIATED PHAGOCYTOSIS + NAFLD | PMID: 32623384, PMID: 34506234 | 91 | 34 | 0.004912 | 0.045681 |

*** “KEGG” – the name of database, was remove from the Google Scholar search term**

**Database VI – Reactome**

| **No.** | **GeneSet** | **Google Scholar search term*** | **Results from Google Scholar searching - PMID** | **Total number of genes related to the “GeneSet” term** | **Number of genes from our input gene set, that overlap with genes related to the “GeneSet” term** | **Enrichment P-value** | **Adjusted**  **P-value** |
| --- | --- | --- | --- | --- | --- | --- | --- |
| 1 | REACTOME SIGNALING BY RHO GTPASES | SIGNALING BY RHO GTPASES + NAFLD | PMID: **27634010,** PMID: **29574534** | 419 | 148 | 6.33E-07 | 0.000949 |
| 2 | REACTOME RHO GTPASE CYCLE | RHO GTPASE CYCLE + NAFLD | PMID: **31467298** | 127 | 56 | 1.38E-06 | 0.001032 |
| 3 | REACTOME MITOTIC SPINDLE CHECKPOINT | MITOTIC SPINDLE CHECKPOINT + NAFLD | No results | 108 | 49 | 2.28E-06 | 0.001102 |
| 4 | REACTOME SIGNALING BY RECEPTOR TYROSINE KINASES | SIGNALING BY RECEPTOR TYROSINE KINASES + NAFLD | PMID: **29038049,**  PMID: **32372975** | 453 | 155 | 2.94E-06 | 0.001102 |
| 5 | REACTOME NEURONAL SYSTEM | NEURONAL SYSTEM + NAFLD | PMID: **24019886** | 389 | 135 | 5.53E-06 | 0.001659 |
| 6 | REACTOME METABOLISM OF LIPIDS | METABOLISM OF LIPIDS + NAFLD | PMID: **33010471,**  PMID: **26198708** | 702 | 224 | 7.21E-06 | 0.001801 |
| 7 | REACTOME DISEASES OF SIGNAL TRANSDUCTION | DISEASES OF SIGNAL TRANSDUCTION + NAFLD | PMID: 19452573,  PMID: 25126158 | 371 | 128 | 1.32E-05 | 0.002561 |
| 8 | REACTOME EFFECTS OF PIP2 HYDROLYSIS | EFFECTS OF PIP2 HYDROLYSIS + NAFLD | PMID: **27132506** | 26 | 17 | 1.37E-05 | 0.002561 |
| 9 | REACTOME MITOTIC PROMETAPHASE | MITOTIC PROMETAPHASE + NAFLD | PMID: **32643289** | 188 | 72 | 2.39E-05 | 0.00398 |
| 10 | REACTOME RESOLUTION OF SISTER CHROMATID COHESION | RESOLUTION OF SISTER CHROMATID COHESION + NAFLD | PMID: **33526312,** PMID: **29974848** | 120 | 50 | 3.24E-05 | 0.004712 |
| 11 | REACTOME SIGNALING BY TGF BETA FAMILY MEMBERS | SIGNALING BY TGF BETA FAMILY MEMBERS + NAFLD | PMID: **30582764,**  PMID: **32054379** | 99 | 43 | 3.46E-05 | 0.004712 |
| 12 | REACTOME SUMOYLATION | SUMOYLATION + NAFLD | PMID: 32710938, PMID: **28094767** | 179 | 68 | 5.33E-05 | 0.006255 |
| 13 | REACTOME SIGNALING BY TGF BETA RECEPTOR COMPLEX | SIGNALING BY TGF BETA RECEPTOR COMPLEX + NAFLD | PMID: **32054379,** PMID: **32191345** | 71 | 33 | 5.42E-05 | 0.006255 |
| 14 | REACTOME NETRIN 1 SIGNALING | NETRIN 1 SIGNALING + NAFLD | PMID: 29985430 | 49 | 25 | 6.44E-05 | 0.006893 |
| 15 | REACTOME PROTEIN PROTEIN INTERACTIONS AT SYNAPSES | PROTEIN PROTEIN INTERACTIONS AT SYNAPSES + NAFLD | PMID: **29564062** | 78 | 35 | 8.06E-05 | 0.008052 |
| 16 | REACTOME CELL CYCLE | CELL CYCLE + NAFLD | PMID: **32578019** | 615 | 193 | 8.74E-05 | 0.008188 |
| 17 | REACTOME NRAGE SIGNALS DEATH THROUGH JNK | NRAGE SIGNALS DEATH THROUGH JNK + NAFLD | Exclude “NRAGE” from search term, result: PMID: **21987620** | 53 | 26 | 0.000108 | 0.009546 |
| 18 | REACTOME VESICLE MEDIATED TRANSPORT | VESICLE MEDIATED TRANSPORT + NAFLD | PMID: 32231001, PMID: 33143043, PMID: 30345152 | 640 | 199 | 0.000122 | 0.010151 |
| 19 | REACTOME P75 NTR RECEPTOR MEDIATED SIGNALLING | P75 NTR RECEPTOR MEDIATED SIGNALLING + NAFLD | PMID: 31296846 | 89 | 38 | 0.000149 | 0.011776 |
| 20 | REACTOME APOPTOTIC CLEAVAGE OF CELLULAR PROTEINS | APOPTOTIC CLEAVAGE OF CELLULAR PROTEINS + NAFLD | PMID: 21476915 | 35 | 19 | 0.000168 | 0.012559 |

*** “REACTOME” – the name of database, was remove from the Google Scholar search term**

**Database VII – GO biological processes**

| **No.** | **GeneSet** | **Google Scholar search term*** | **Results from Google Scholar searching - PMID** | **Total number of genes related to the “GeneSet” term** | **Number of genes from our input gene set, that overlap with genes related to the “GeneSet” term** | **Enrichment P-value** | **Adjusted**  **P-value** |
| --- | --- | --- | --- | --- | --- | --- | --- |
| 1 | GO CELL PROJECTION ORGANIZATION | CELL PROJECTION ORGANIZATION + NAFLD | no results | 1425 | 494 | 1.69E-18 | 1.24E-14 |
| 2 | GO BIOLOGICAL ADHESION | BIOLOGICAL ADHESION + NAFLD | PMID: **23913408** | 1342 | 456 | 2.06E-15 | 7.58E-12 |
| 3 | GO NEUROGENESIS | NEUROGENESIS + NAFLD | PMID: 22280920 | 1521 | 497 | 2.13E-13 | 5.21E-10 |
| 4 | GO CELLULAR COMPONENT MORPHOGENESIS | CELLULAR COMPONENT MORPHOGENESIS + NAFLD | PMID: 34353365 | 1056 | 362 | 5.39E-13 | 9.90E-10 |
| 5 | GO NEURON DEVELOPMENT | NEURON DEVELOPMENT + NAFLD | PMID: **20413174** | 1040 | 354 | 2.83E-12 | 3.77E-09 |
| 6 | GO REGULATION OF SMALL GTPASE MEDIATED SIGNAL TRANSDUCTION | REGULATION OF SMALL GTPASE MEDIATED SIGNAL TRANSDUCTION + NAFLD | PMID: **32139507** | 310 | 132 | 3.08E-12 | 3.77E-09 |
| 7 | GO SMALL GTPASE MEDIATED SIGNAL TRANSDUCTION | SMALL GTPASE MEDIATED SIGNAL TRANSDUCTION + NAFLD | PMID: **32139507** | 516 | 197 | 4.87E-12 | 5.12E-09 |
| 8 | GO NEURON DIFFERENTIATION | NEURON DIFFERENTIATION + NAFLD | No results, however this term is associated with nervous system | 1276 | 420 | 5.96E-12 | 5.48E-09 |
| 9 | GO ADHERENS JUNCTION ORGANIZATION | ADHERENS JUNCTION ORGANIZATION + NAFLD | PMID: 24966608, PMID: 30453660 | 137 | 69 | 7.91E-11 | 6.46E-08 |
| 10 | GO REGULATION OF CELL PROJECTION ORGANIZATION | REGULATION OF CELL PROJECTION ORGANIZATION + NAFLD | no results | 635 | 227 | 1.99E-10 | 1.46E-07 |
| 11 | GO REGULATION OF NERVOUS SYSTEM DEVELOPMENT | REGULATION OF NERVOUS SYSTEM DEVELOPMENT + NAFLD | PMID: **34256416** | 870 | 295 | 3.23E-10 | 2.00E-07 |
| 12 | GO CELL PART MORPHOGENESIS | CELL PART MORPHOGENESIS + NAFLD | PMID: 34353365 | 638 | 227 | 3.26E-10 | 2.00E-07 |
| 13 | GO REGULATION OF CELL MORPHOGENESIS | REGULATION OF CELL MORPHOGENESIS + NAFLD | PMID: 34353365 | 448 | 168 | 8.83E-10 | 4.99E-07 |
| 14 | GO CELLULAR RESPONSE TO ENDOGENOUS STIMULUS | CELLULAR RESPONSE TO ENDOGENOUS STIMULUS + NAFLD | No results | 1312 | 417 | 1.33E-09 | 7.00E-07 |
| 15 | GO REGULATION OF CELLULAR COMPONENT BIOGENESIS | REGULATION OF CELLULAR COMPONENT BIOGENESIS + NAFLD | PMID: **32597705** | 858 | 288 | 1.70E-09 | 7.41E-07 |
| 16 | GO REGULATION OF CELL DEVELOPMENT | REGULATION OF CELL DEVELOPMENT + NAFLD | PMID: 32578019 | 893 | 298 | 1.75E-09 | 7.41E-07 |
| 17 | GO DENDRITE DEVELOPMENT | DENDRITE DEVELOPMENT + NAFLD | PMID: 26339640 | 203 | 89 | 1.79E-09 | 7.41E-07 |
| 18 | GO CELL JUNCTION ORGANIZATION | CELL JUNCTION ORGANIZATION + NAFLD | PMID: **34280073,** PMID: **30670819** | 276 | 113 | 1.81E-09 | 7.41E-07 |
| 19 | GO REGULATION OF RAS PROTEIN SIGNAL TRANSDUCTION | REGULATION OF RAS PROTEIN SIGNAL TRANSDUCTION + NAFLD | No results, however term: “RAS + NAFLD”, gave result: PMID: 30401637 | 219 | 94 | 2.42E-09 | 9.35E-07 |
| 20 | GO REGULATION OF NEURON PROJECTION DEVELOPMENT | REGULATION OF NEURON PROJECTION DEVELOPMENT + NAFLD | No results | 465 | 171 | 3.38E-09 | 1.24E-06 |

*** “GO” – the name of database, was remove from the Google Scholar search term**

**Database VIII – GO cellular components**

| **No.** | **GeneSet** | **Google Scholar search term*** | **Results from Google Scholar searching - PMID** | **Total number of genes related to the “GeneSet” term** | **Number of genes from our input gene set, that overlap with genes related to the “GeneSet” term** | **Enrichment P-value** | **Adjusted**  **P-value** |
| --- | --- | --- | --- | --- | --- | --- | --- |
| 1 | GO NEURON PART | NEURON PART + NAFLD | PMID: **27273168** | 1618 | 560 | 9.05E-21 | 9.06E-18 |
| 2 | GO SYNAPSE | SYNAPSE + NAFLD | PMID: **32708059** | 1113 | 400 | 5.79E-18 | 2.90E-15 |
| 3 | GO CELL PROJECTION PART | CELL PROJECTION PART + NAFLD | no results | 1345 | 467 | 1.18E-17 | 3.95E-15 |
| 4 | GO CELL JUNCTION | CELL JUNCTION + NAFLD | PMID: **30591521,** PMID: **27273168** | 1212 | 419 | 1.47E-15 | 3.68E-13 |
| 5 | GO MICROTUBULE CYTOSKELETON | MICROTUBULE CYTOSKELETON + NAFLD | https://doi.org/10.1016/j.livres.2018.09.003,  PMID: **21476912** | 1096 | 384 | 2.59E-15 | 5.19E-13 |
| 6 | GO NEURON PROJECTION | NEURON PROJECTION + NAFLD | PMID: **20413174** | 1223 | 419 | 7.49E-15 | 1.25E-12 |
| 7 | GO CYTOSKELETAL PART | CYTOSKELETAL PART + NAFLD | PMID: 28652891 | 1545 | 505 | 1.25E-13 | 1.79E-11 |
| 8 | GO MICROTUBULE ORGANIZING CENTER | MICROTUBULE ORGANIZING CENTER + NAFLD | No results | 684 | 254 | 1.48E-13 | 1.85E-11 |
| 9 | GO SYNAPSE PART | SYNAPSE PART + NAFLD | PMID: **32068022** | 888 | 307 | 1.14E-11 | 1.27E-09 |
| 10 | GO ANCHORING JUNCTION | ANCHORING JUNCTION + NAFLD | PMID: 25400436 | 530 | 198 | 4.08E-11 | 4.09E-09 |
| 11 | GO SOMATODENDRITIC COMPARTMENT | SOMATODENDRITIC COMPARTMENT + NAFLD | No results, however this term is associated with nervous system | 773 | 268 | 1.76E-10 | 1.60E-08 |
| 12 | GO CELL LEADING EDGE | CELL LEADING EDGE + NAFLD | No results | 381 | 149 | 2.71E-10 | 2.18E-08 |
| 13 | GO CENTROSOME | CENTROSOME + NAFLD | PMID: **32623384** | 478 | 179 | 2.83E-10 | 2.18E-08 |
| 14 | GO DENDRITIC TREE | DENDRITIC TREE + NAFLD | No results, however this term is associated with nervous system | 552 | 199 | 1.20E-09 | 8.57E-08 |
| 15 | GO GOLGI APPARATUS | GOLGI APPARATUS + NAFLD | PMID: **32920226, PMID: 32444289** | 1430 | 449 | 1.79E-09 | 1.20E-07 |
| 16 | GO POSTSYNAPSE | POSTSYNAPSE + NAFLD | PMID: **31338065** | 580 | 206 | 2.55E-09 | 1.60E-07 |
| 17 | GO NEURON TO NEURON SYNAPSE | NEURON TO NEURON SYNAPSE + NAFLD | PMID: **32068022** | 327 | 128 | 4.51E-09 | 2.64E-07 |
| 18 | GO PLASMA MEMBRANE REGION | PLASMA MEMBRANE REGION + NAFLD | PMID: **29059699,** PMID: **32978374** | 1127 | 362 | 4.75E-09 | 2.64E-07 |
| 19 | GO CYTOPLASMIC REGION | CYTOPLASMIC REGION + NAFLD | PMID: **23840612** | 465 | 169 | 1.10E-08 | 5.80E-07 |
| 20 | GO CELL CELL JUNCTION | CELL CELL JUNCTION + NAFLD | PMID: **30591521,** PMID: **27273168** | 425 | 155 | 3.40E-08 | 1.70E-06 |

*** “GO” – the name of database, was remove from the Google Scholar search term**

**Database IX – GO molecular functions**

| **No.** | **GeneSet** | **Google Scholar search term*** | **Results from Google Scholar searching - PMID** | **Total number of genes related to the “GeneSet” term** | **Number of genes from our input gene set, that overlap with genes related to the “GeneSet” term** | **Enrichment P-value** | **Adjusted**  **P-value** |
| --- | --- | --- | --- | --- | --- | --- | --- |
| 1 | GO ADENYL NUCLEOTIDE BINDING | ADENYL NUCLEOTIDE BINDING + NAFLD | No results | 1457 | 504 | 1.17E-18 | 1.93E-15 |
| 2 | GO CYTOSKELETAL PROTEIN BINDING | CYTOSKELETAL PROTEIN BINDING + NAFLD | PMID: **23913408** | 902 | 335 | 1.53E-17 | 1.26E-14 |
| 3 | GO RIBONUCLEOTIDE BINDING | RIBONUCLEOTIDE BINDING + NAFLD | PMID: 33927635 | 1779 | 587 | 1.09E-16 | 5.97E-14 |
| 4 | GO DRUG BINDING | DRUG BINDING + NAFLD | PMID: **30194708** | 1622 | 532 | 1.26E-14 | 5.16E-12 |
| 5 | GO GTPASE BINDING | GTPASE BINDING + NAFLD | PMID: **32139507** | 490 | 187 | 1.77E-11 | 5.82E-09 |
| 6 | GO PROTEIN SERINE THREONINE KINASE ACTIVITY | PROTEIN SERINE THREONINE KINASE ACTIVITY + NAFLD | PMID: **29404514** | 411 | 162 | 2.21E-11 | 6.05E-09 |
| 7 | GO KINASE ACTIVITY | KINASE ACTIVITY + NAFLD | PMID: **31760070** | 714 | 254 | 2.89E-11 | 6.78E-09 |
| 8 | GO PROTEIN KINASE ACTIVITY | PROTEIN KINASE ACTIVITY + NAFLD | PMID: **34278168**, PMID: **31760070** | 559 | 205 | 1.24E-10 | 2.55E-08 |
| 9 | GO GUANYL NUCLEOTIDE EXCHANGE FACTOR ACTIVITY | GUANYL NUCLEOTIDE EXCHANGE FACTOR ACTIVITY + NAFLD | PMID: **15331065** | 201 | 91 | 1.52E-10 | 2.78E-08 |
| 10 | GO TRANSFERASE ACTIVITY TRANSFERRING PHOSPHORUS CONTAINING GROUPS | TRANSFERASE ACTIVITY TRANSFERRING PHOSPHORUS CONTAINING GROUPS + NAFLD | No results | 857 | 292 | 2.23E-10 | 3.67E-08 |
| 11 | GO PROTEIN DOMAIN SPECIFIC BINDING | PROTEIN DOMAIN SPECIFIC BINDING + NAFLD | PMID: **19352614** | 660 | 233 | 4.35E-10 | 6.50E-08 |
| 12 | GO SMALL GTPASE BINDING | SMALL GTPASE BINDING + NAFLD | PMID: **21703172** | 399 | 153 | 8.17E-10 | 1.12E-07 |
| 13 | GO ACTIN BINDING | ACTIN BINDING + NAFLD | PMID: **23545492** | 398 | 151 | 2.48E-09 | 3.07E-07 |
| 14 | GO ATPASE ACTIVITY | ATPASE ACTIVITY + NAFLD | PMID: **29220698** | 421 | 158 | 2.61E-09 | 3.07E-07 |
| 15 | GO RAS GUANYL NUCLEOTIDE EXCHANGE FACTOR ACTIVITY | RAS GUANYL NUCLEOTIDE EXCHANGE FACTOR ACTIVITY + NAFLD | PMID: **29464180** | 130 | 63 | 3.75E-09 | 4.12E-07 |
| 16 | GO PHOSPHATIDYLINOSITOL BINDING | PHOSPHATIDYLINOSITOL BINDING + NAFLD | PMID: 23431468 | 229 | 95 | 1.59E-08 | 1.64E-06 |
| 17 | GO RHO GUANYL NUCLEOTIDE EXCHANGE FACTOR ACTIVITY | RHO GUANYL NUCLEOTIDE EXCHANGE FACTOR ACTIVITY + NAFLD | No results, however the term “RHO + NAFLD” give result: PMID: 34610720 | 73 | 40 | 3.59E-08 | 3.47E-06 |
| 18 | GO ATPASE ACTIVITY COUPLED | ATPASE ACTIVITY COUPLED + NAFLD | PMID: **29220698,**  PMID: 30171159 | 342 | 129 | 5.24E-08 | 4.79E-06 |
| 19 | GO HYDROLASE ACTIVITY ACTING ON ACID ANHYDRIDES | HYDROLASE ACTIVITY ACTING ON ACID ANHYDRIDES + NAFLD | No results | 873 | 282 | 1.46E-07 | 1.26E-05 |
| 20 | GO SMAD BINDING | SMAD BINDING + NAFLD | PMID: **34280515** | 76 | 39 | 5.28E-07 | 4.34E-05 |

*** “GO” – the name of database, was remove from the Google Scholar search term**

**References**

1. Thompson FE, Subar AF. Chapter 1 - Dietary Assessment Methodology. In: Coulston AM, Boushey CJ, Ferruzzi MG, Delahanty LM, editors. Nutrition in the Prevention and Treatment of Disease (Fourth Edition): Academic Press; 2017. p. 5-48.

2. Willett WC, Howe GR, Kushi LH. Adjustment for total energy intake in epidemiologic studies. Am J Clin Nutr. 1997;65(4 Suppl):1220S-8S; discussion 9S-31S.

3. Cade JE, Burley VJ, Warm DL, Thompson RL, Margetts BM. Food-frequency questionnaires: a review of their design, validation and utilisation. Nutr Res Rev. 2004;17(1):5-22.

4. Trichopoulou A, Kouris-Blazos A, Wahlqvist ML, Gnardellis C, Lagiou P, Polychronopoulos E, et al. Diet and overall survival in elderly people. BMJ. 1995;311(7018):1457-60.

5. Craig CL, Marshall AL, Sjostrom M, Bauman AE, Booth ML, Ainsworth BE, et al. International physical activity questionnaire: 12-country reliability and validity. Med Sci Sports Exerc. 2003;35(8):1381-95.

6. Lear SA, Hu W, Rangarajan S, Gasevic D, Leong D, Iqbal R, et al. The effect of physical activity on mortality and cardiovascular disease in 130 000 people from 17 high-income, middle-income, and low-income countries: the PURE study. Lancet. 2017;390(10113):2643-54.

7. Skonieczna-Zydecka K, Grochans E, Maciejewska D, Szkup M, Schneider-Matyka D, Jurczak A, et al. Faecal Short Chain Fatty Acids Profile is Changed in Polish Depressive Women. Nutrients. 2018;10(12).

8. Pawlik A, Baskiewicz-Masiuk M, Machalinski B, Gawronska-Szklarz B. The effect of methotrexate and glucocorticosteroids on apoptosis of phythemaglutinin-stimulated mononuclear cells from peripheral blood. Fundam Clin Pharmacol. 2005;19(1):81-5.

9. Archin NM, Bateson R, Tripathy MK, Crooks AM, Yang KH, Dahl NP, et al. HIV-1 expression within resting CD4+ T cells after multiple doses of vorinostat. J Infect Dis. 2014;210(5):728-35.

10. Dispirito JR, Shen H. Histone acetylation at the single-cell level: a marker of memory CD8+ T cell differentiation and functionality. J Immunol. 2010;184(9):4631-6.

11. Giraldo AM, Lynn JW, Purpera MN, Godke RA, Bondioli KR. DNA methylation and histone acetylation patterns in cultured bovine fibroblasts for nuclear transfer. Mol Reprod Dev. 2007;74(12):1514-24.

12. Mansell G, Gorrie-Stone TJ, Bao Y, Kumari M, Schalkwyk LS, Mill J, et al. Guidance for DNA methylation studies: statistical insights from the Illumina EPIC array. BMC Genomics. 2019;20(1):366.

13. Fowlkes EB, Mallows CL. A Method for Comparing Two Hierarchical Clusterings. Journal of the American Statistical Association. 1983;78(383):553-69.

14. Le TT, Fu W, Moore JH. Scaling tree-based automated machine learning to biomedical big data with a feature set selector. Bioinformatics. 2020;36(1):250-6.

15. Oki S, Ohta T, Shioi G, Hatanaka H, Ogasawara O, Okuda Y, et al. ChIP-Atlas: a data-mining suite powered by full integration of public ChIP-seq data. EMBO Rep. 2018;19(12).

16. Heinz S, Benner C, Spann N, Bertolino E, Lin YC, Laslo P, et al. Simple combinations of lineage-determining transcription factors prime cis-regulatory elements required for macrophage and B cell identities. Mol Cell. 2010;38(4):576-89.
